# The dietary changes during Ramadan and their impact on anthropometry, blood pressure, and metabolic profile

**DOI:** 10.3389/fnut.2024.1394673

**Published:** 2024-06-10

**Authors:** Rami Al-Jafar, Wang Yuqi, Paul Elliott, Konstantinos K. Tsilidis, Abbas Dehghan

**Affiliations:** ^1^Department of Epidemiology and Biostatistics, School of Public Health, Imperial College London, London, United Kingdom; ^2^Data Services Sector, Lean Business Services, Riyadh, Saudi Arabia; ^3^Dementia Research Institute at Imperial College London, London, United Kingdom; ^4^National Institute for Health Research Imperial College Biomedical Research Centre, Imperial College London, London, United Kingdom; ^5^Department of Hygiene and Epidemiology, University of Ioannina School of Medicine, Ioannina, Greece; ^6^MRC-PHE Centre for Environment and Health, School of Public Health, Imperial College London, London, United Kingdom

**Keywords:** food intake, nutrients, metabolomics, intermittent fasting, dietary transition

## Abstract

**Background:**

The effect of Ramadan intermittent fasting (RIF) on the metabolic profile, anthropometry and blood pressure has been investigated in multiple studies. However, it is still unknown to what extent changes in nutrient intakes contribute to these changes.

**Methods:**

This observational study was conducted in London (UK) in 2019. The study collected diverse data from a community-based sample in London before and during/after Ramadan. Collected data included a 3-day food diary (before and during Ramadan), as well as blood samples, anthropometric measurements and blood pressure (before and after Ramadan). The food diary was translated into nutritional data using nutrition software “Nutritics.” The changes in nutrient intakes were investigated using a mixed-effects regression model. The impact of adjusting for nutrient intake change was investigated on the absolute difference of metabolites (Nightingale platform), systolic/diastolic blood pressure and anthropometric measures.

**Results:**

The study collected data on food intake before and during Ramadan from 56 participants; the mean age was 44.7 ± 17.3, and 51.8% (*n* = 29) were females. We found a change in the intake of 11 nutritional factors, glucose, fructose, betaine, sugars, sugars as monosaccharide equivalents, lutein/zeaxanthin, starch, starch as monosaccharide equivalents, proline, glutamic acid and lycopene. No changes in quantities or proportions of macronutrients, carbohydrates, protein and fat. Mainly, the changes in diet during Ramadan are characterized by more consumption of sugars (62%, *p* < 0.001) and a lower intake of starch (−21%, *p* = 0.012). The changes in 14 metabolite levels (two glycolysis-related metabolites, one amino acid, two ketone bodies, two triglyceride, six lipoprotein subclasses, and an inflammation marker) after Ramadan were partially associated with some changes in nutrient intakes during Ramadan, especially betaine, fructose, glucose, starches and sugars. The lutein/zeaxanthin intake change explained inversely 14% of systolic blood pressure changes. Moreover, BMI and weight changes were partially explained by changes in intake of fat (7%; 9%), monounsaturated fat (6%; 7%), starch (8%; 9%), and starch as monosaccharide equivalents (8%; 9%) intakes in a direct relationship.

**Conclusion:**

Diet changes during Ramadan were associated partially with the observed changes in the metabolic profile, blood pressure and anthropometry. This confirms the changes associated with RIF in the metabolic profile, blood pressure and anthropometry are not an absolute physiological response to the diet transition occurring during Ramadan.

## 1 Introduction

Ramadan intermittent fasting (RIF) is associated with a sudden drastic change in the dietary behavior of observers. Although observing RIF does not dictate restriction on calorie intake or type of food, previous studies showed that it incurs significant changes in eating habits, patterns, duration, frequency of meals and intakes of energy and macronutrients (1–4). Observers feast after sunset (Iftar) and eat another lighter meal before dusk (Suhur) (5) and more often in groups of family and friends (6). These changes, along with changes in sleep duration and quality (7), circadian rhythm (8) and physical activity (9), affect cardiometabolic risk markers (10) and inflammatory and oxidative stress markers (11).

Understanding these dietary changes during Ramadan is crucial for developing evidence-based guidelines for the public and healthcare professionals. This is particularly important for individuals with metabolic disorders such as diabetes or hypertension who want to practice this ritual safely.

Previous studies have reported inconsistent results with regards to energy intake ranging from an increase (12, 13), to no change (14, 15) or even a reduction (16–18) during Ramadan. Likewise, reported studies are inconsistent on the intake of macronutrients, including fat (3, 14, 19), carbohydrates (13, 17, 19), cholesterol (13, 14), and protein (3, 19). Recently, Abdelrahim et al. conducted a meta-analysis and reported a reduction in total energy intake, carbohydrates, protein and water intakes and no change in fat or protein intakes during Ramadan (4). The previous observational studies often focused on specific ethnic groups or nationalities, potentially skewing the results. Therefore, investigating these changes in a multi-ethnic sample is essential to minimize cultural bias.

Moreover, most previous studies used 24-h dietary recalls (14, 20) and food frequency questionnaires (17, 18) to measure diet for their ease of use, which are subject to recall bias. Food diary is a cheap and convenient real-time recording method that provides a more accurate measure of diet (21).

In addition, former studies have not investigated to what extent the changes in dietary intake during Ramadan explain the subsequent changes in metabolites, systolic/diastolic blood pressure and anthropometric indices. Investigating this will allow more structured nutritional advice to Ramadan observers, especially individuals with chronic diseases. We hypothesize that diet changes during Ramadan are associated with the changes observed after Ramadan in blood pressure, anthropometry and metabolic profile.

This study aims to explore changes in energy, macronutrient and nutritional factors intakes during Ramadan in a multi-ethnic population. It will investigate whether these dietary changes explain the changes in the metabolic profile, blood pressure and anthropometric indices after Ramadan. The findings could contribute to a better understanding of the health impacts of Ramadan fasting.

## 2 Methods

LORANS is an observational study conducted to observe the effect of RIF on cardiometabolic health in 2019. The study was conducted in five large mosques in London, UK; details of the study are described elsewhere (22). In LORANS, participants were given a 3-day food diary to monitor the change in their dietary habits during Ramadan. The food diary had an introduction illustrating how to describe types/amounts of food and two sections to record food intake 3 days before and 3 days during RIF. An example of 1 day’s food intake was given to show the participant how to fill in food intake accurately. The 3 days consisted of one weekend day and two weekdays to ensure we captured a holistic picture of dietary habits. Each food diary was labeled with a unique barcode to identify the participant (Supplementary material 1). In LORANS, we collected diverse data from 146 participants who attended the first visit before Ramadan to participate in the study. Of those, 56 participants (response rate = 38.5%) agreed to fill out the food diary and returned it completed on the second visit after Ramadan.

We used “Nutritics,” a nutrition software, to translate the food diaries into nutritional data (23). The platform output provided the intakes of macronutrients and nutritional factors. However, the energy value from each macronutrient was not provided, so some extra calculations were needed. Our estimation of the macronutrients’ energy values was based on the fact that 1 g of protein, fat and carbs are 4 kcal, 9 kcal and 4 kcal, respectively (24).

As described previously (22, 25, 26), we measured systolic blood pressure (SBP), diastolic blood pressure (DBP), weight, waist circumference (WC), hip circumference (HC), body mass index (BMI), fat mass (FM), fat percentage (F%), fat-free mass (FFM), and total body water (TBW). Also, we collected two blood samples from LORANS participants a few days before and 8–12 days after Ramadan. Metabolomic profiling was performed using nuclear magnetic resonance (NMR) spectroscopy to assess metabolite changes.

### 2.1 Statistical analysis

Considering fat as the primary outcome, we calculated that based on a significant level of 0.05 and 80% power, we need to collect before and after Ramadan food intake data from 25 subjects to detect a significant difference of 5%. Using the “lme4” package in R (version 4.1.0), we constructed mixed-effects models adjusted for fixed (age and sex) and random variables (mosque) to estimate the transition in diet during Ramadan. In addition, we applied a False Discovery Rate (FDR) method to correct for multiple testing. The same test was applied to metabolites for men and women separately to investigate whether the effect of RIF on metabolites varies between men and women and impacts the metabolomic changes upon the observance of RIF. This analysis was performed using the “lmer” package in R. We presented all variables in the form of mean ± SD before and during Ramadan and all results as a mean difference with 95% CI. Results with an FDR-adjusted *p*-value of less than 0.05 were considered statistically significant.

To explore to what extent the changes in diet explain the changes in metabolites, we calculated the absolute differences in nutrients (intake during minus intake before Ramadan) and metabolites (before and after Ramadan) that changed significantly. Afterwards, each metabolite’s absolute difference was added to a basic linear regression model as a dependent variable with age, sex, number of fasting days and mosque as independent variables, and the R-squared value was reported. Then, a nutrient was added to the same model to report the change in the R-squared value (compared to the original R-squared value in the basic model), which was interpreted as the proportion of the metabolite’s change explained by the change in the nutrient intakes. The same process was repeated for SBP, DBP, weight, WC, HC, BMI, FM, F%, FFM, and TBW as dependent variables that changed after RIF (22, 25).

## 3 Results

The mean ± SD age of the 56 participants who completed the food diaries before and during Ramadan was 44.7 ± 17.3, and 29 (51.8%) of them were females. Table 1 shows the baseline characteristics of this group.

**Table 1:** Baseline characteristics of LORANS’s participants who filled out and returned the food diary (*n* = 56).

Variable	Sub-groups	Value
Age (mean ± SD)	Total	44.7 ± 17.3
	18–40 years (%)	37.5%
	40–60 years (%)	42.8%
	60–80 years (%)	17.9%
	> 80 years (%)	1.8%
Sex (number of females (%))	*n* = 29 (51.8%)	
Ethnic background (%)	South Asian	59%
	Arab	22%
	Other	14%
	Unknown	5%
Marital status (%)	Single	23.2%
	Married/living with a partner	67.9%
	Divorced/separated	3.5%
	Unknown	5.4%
With chronic diseases (%)	Diabetes	8.9%
	Hypertension	19.6%
	Cardiovascular diseases	1.8%
Education (%)	No formal qualification	8.9%
	Secondary school or equivalent	26.8%
	Higher education: College/HNC/HND	17.9%
	Bachelor’s degree	26.8%
	Postgraduate degree	14.3%
	Unknown	5.3%
Smoking (%)	Never	83.9%
	Stopped	11.6%
	Occasionally	0.9%
	Yes, most or all days	3.6%

During Ramadan, there were significant changes in the intake of 11 nutritional factors. Table 2 indicates that RIF is mainly characterized by the consumption of more sugars and a lower intake of starch. However, there was no change in the quantities or proportions of macronutrients (Table 3).

**Table 2:** Before and after Ramadan intakes of nutritional factors (*n* = 11), whose intakes significantly changed during Ramadan, besides some essential nutrients whose intakes did not change (*n* = 6).

Nutrient	Intake before Ramadan mean (SD)	Intake during Ramadan mean (SD)	Mean difference (95% CI)	Percentage of change	FDR-adjusted *p*-value
Glucose (g)	8.6 (10)	27.2 (17)	18.6 (14.2 to 23)	216%	<0.001
Fructose (g)	10.2 (11)	28.1 (17)	18 (13.5 to 22.4)	175%	<0.001
Betaine (mg)	0 (0)	0.2 (0)	0.2 (0.15 to 0.26)	NA	<0.001
Sugars (g)	58.7 (29)	95.2 (47)	36.5 (25.7 to 47.3)	62%	<0.001
Sugars as monosaccharide equivalents (g)	57.1 (30)	92 (48)	34.9 (23.4 to 46.4)	61%	<0.001
Lutein/Zeaxanthin (mg)	2.2 (9)	12.9 (11)	−10.7 (−14.2 to −7.2)	486%	<0.001
Starch (g)	116.1 (41)	91.7 (41)	−24.4 (−37.1 to −11.7)	−21%	0.012
Starch as monosaccharide equivalents (g)	121.9 (44)	95 (46)	−26.9 (−41 to −12.8)	−22%	0.012
Proline (mg)	2,740 (1688)	1986 (1098)	−754 (−1,176 to −332)	−28%	0.022
Glutamic acid (mg)	8,379 (5653)	5,890 (3388)	−2,489 (−3951.9 to −1026.3)	−30%	0.032
Lycopene (mg)	830 (2339)	3,886 (7089)	3,056 (1,241 to 4,871)	368%	0.033
Potassium (mg)	2104.4 (725)	2343.5 (820)	239 (64 to 414)	11%	0.087
Sodium (mg)	1,431 (572)	1291.4 (684)	−139 (−330 to 51)	−10%	0.344
Magnesium (mg)	200.1 (71)	210.2 (75)	10.2 (−7.8 to 28.2)	5%	0.488
Cholesterol (mg)	204.5 (165)	181.9 (126)	−22.6 (−63 to 17.4)	−11%	0.488
Fibre (g)	15.9 (6)	16.6 (6)	0.7 (−0.6 to 2)	4%	0.521
Calcium (mg)	529.2 (221)	523.2 (261)	−6 (−75 to 63)	−1%	0.999

**Table 3:** Changes in energy and macronutrients during Ramadan compared to regular diet before Ramadan.

Nutrient	Intake before Ramadan mean (SD)	Intake during Ramadan mean (SD)	Mean difference (95% CI)	Percentage of change	FDR-adjusted *p*-value
Energy (kcal)	1526.7 (487)	1504.2 (577)	4.2 (−115 to 123)	0%	0.973
Carbohydrates					
(g)	176.4 (58)	192 (72)	16.9 (0.3 to 34)	10%	0.214
% of energy	47.1 (9)	51.9 (9)	4.4 (1.3 to 7.5)	9%	0.079
Protein					
(g)	70.8 (34)	62.1 (25)	−7.4 (−16.1 to 1.3)	−10%	0.292
% of energy	18.5 (6)	16.8 (4)	−1.7 (−3.2 to −0.1)	−9%	0.196
Fat					
(g)	59.7 (25)	54.3 (29)	−3.8 (−10.4 to 3)	−6%	0.506
% of energy	34.1 (7)	31.2 (8)	−2.6 (−5 to −0.1)	−8%	0.208
Saturated fat (g)	20.1 (9)	18 (11)	−1.7 (−4.5 to 1.1)	−8%	0.473
Monounsaturated fat (g)	18.5 (9)	16.5 (9)	−1.3 (−3.6 to 0.9)	−7%	0.473
Polyunsaturated fat (g)	8.5 (5)	7.8 (4)	−0.4 (−1.6 to 0.9)	−4%	0.715

We reported significant changes in 78 metabolites in women, while only one metabolite changed in men (Supplementary materials 2, 3). When investigating metabolite changes based on having a chronic disease (with/without a chronic disease), no changes were observed in the two groups’ metabolites (Supplementary materials 4, 5).

The key nutrients that were associated with the metabolite changes were lutein/zeaxanthin, lycopene, fiber, fructose, glucose, starch, starch as monosaccharide equivalents, sugars and sugars as monosaccharide equivalents. Notably, the most substantial changes in metabolites, as explained by alterations in nutrient intake, were observed in large high-density lipoprotein (HDL) with 25% explained by alteration in glucose intake followed by.

**Figure 1:**
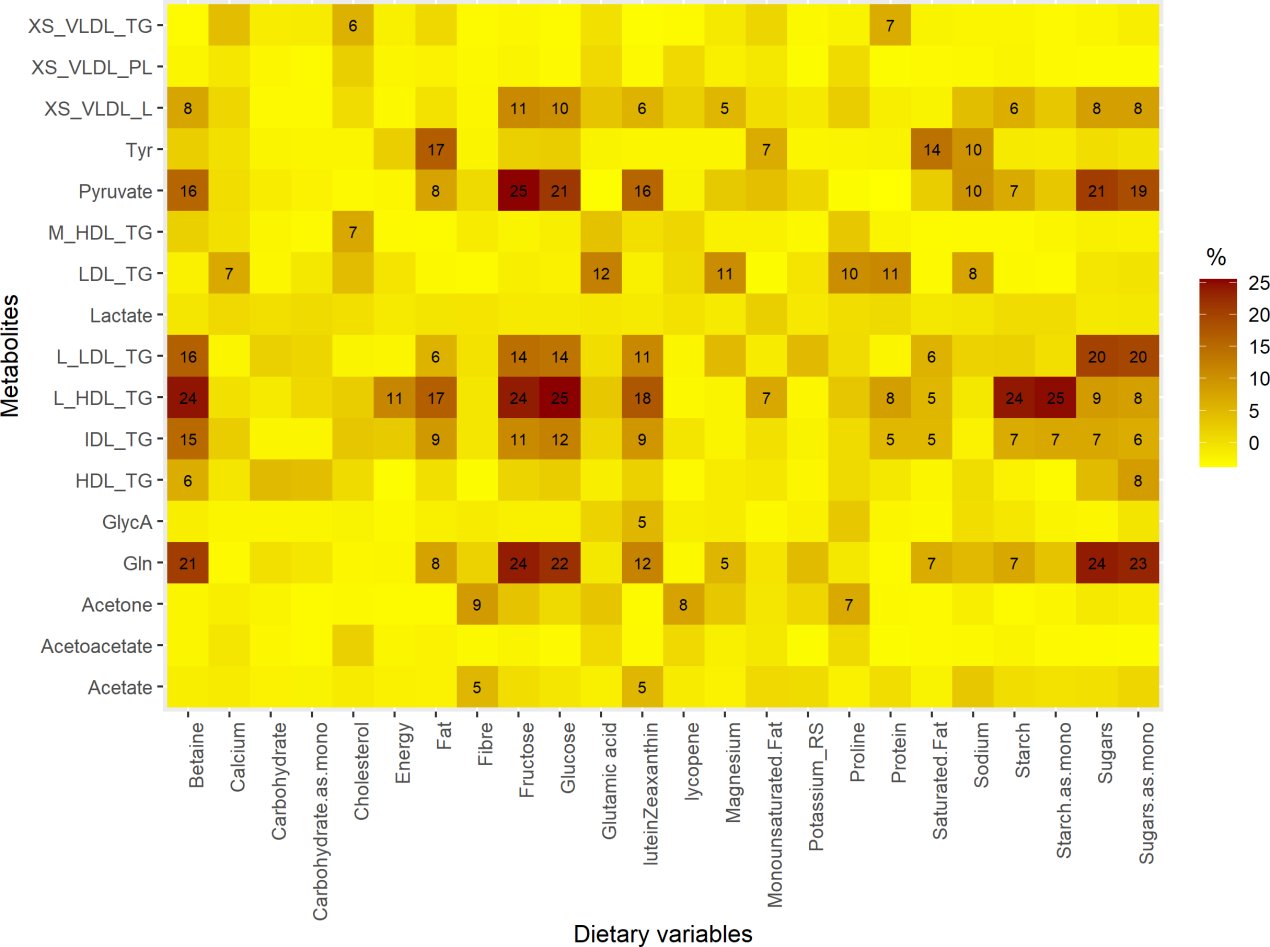
The proportions of metabolites explained by changes in intakes of dietary variables. Gln, Glutamine; GlycA, Glycoprotein acetyls; L_HDL_TG, Triglycerides in large HDL; IDL_TG, Triglycerides in IDL; M_HDL_TG, Triglycerides in medium HDL; L_LDL_TG, Triglycerides in large LDL; HDL_TG, Triglycerides in HDL; Tyr, Tyrosine; LDL_TG, Triglycerides in LDL; XS_VLDL_PL, Phospholipids in very small VLDL; XS_VLDL_TG, Triglycerides in very small VLDL. The metabolites are those that changed significantly after Ramadan. The dietary variables are those whose intake changed significantly during Ramadan, macronutrients, energy, potassium, sodium, magnesium, cholesterol, fiber and calcium. Mono, monosaccharide equivalents.

Starch as monosaccharide (25%), starch (24%), fructose (24%) and betaine (24%). Similarly, 25% of pyruvate change was explained by fructose intake alteration followed by glucose (21%), and sugar (21%). Also, fructose and sugar intake alterations were deemed to explain changes in glutamine by 24 and 24%, respectively (Figure 1).

**Figure 2:**
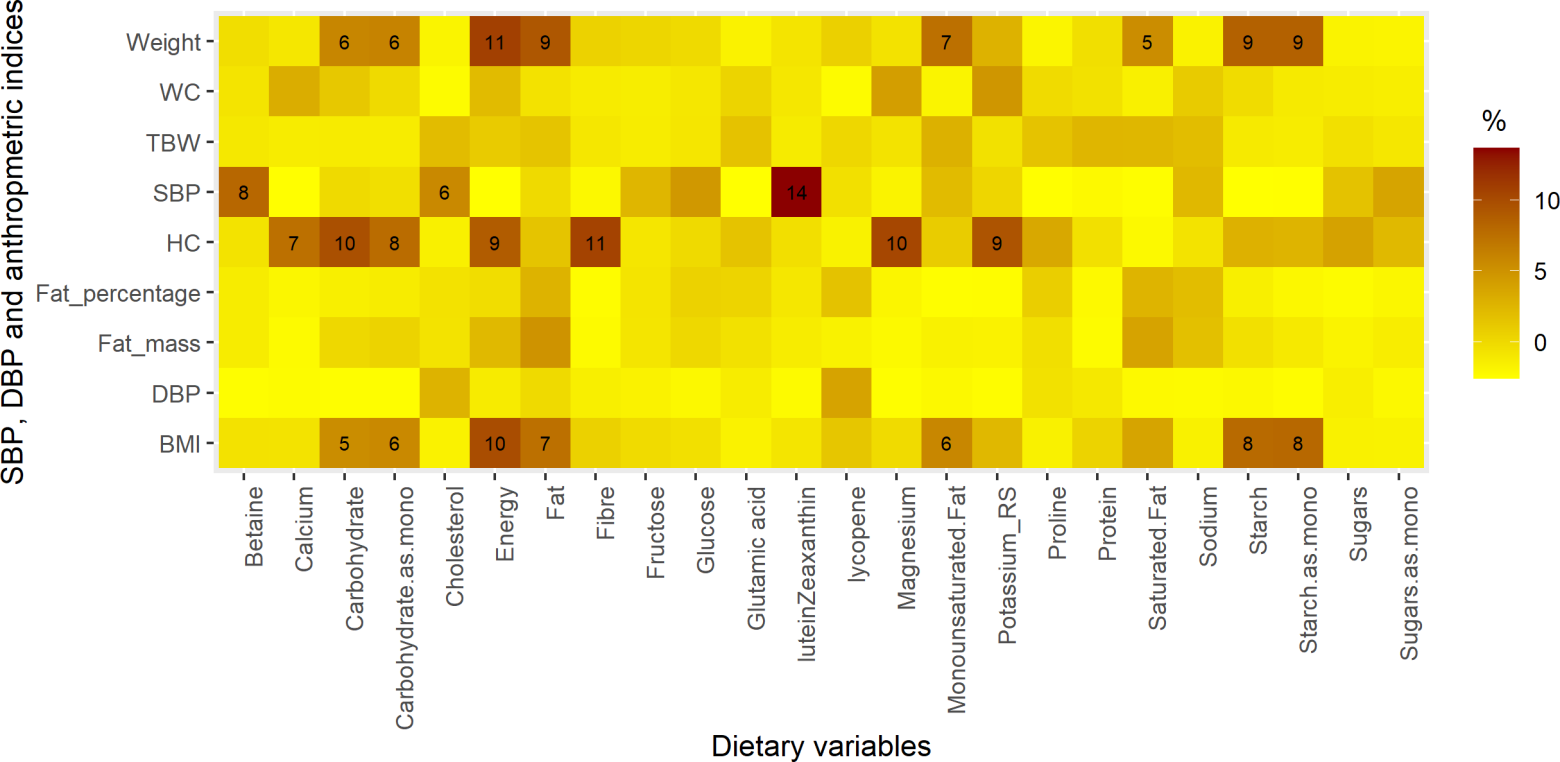
The proportions of blood pressure and anthropometric parameters explained by changes in intakes of dietary variables. The dietary variables are those whose intake changed significantly during Ramadan, macronutrients, energy, potassium, sodium, magnesium, cholesterol, fiber and calcium.

SBP, DBP, weight, WC, HC, BMI, FM, F%, and TBW were less affected by changes in nutrient intakes. The largest influence was reported for SBP, where 14% of the change was explained by the change in Lutein/Zeaxanthin intake. Additionally, changes in the intake of carbohydrates (and their monosaccharide equivalents), fat, monounsaturated fat, and starch (and their monosaccharide equivalent) were associated with changes in weight and BMI (association range from 5 to 9%) (Figure 2).

## 4 Discussion

### 4.1 Principal findings

In a general population from different ethnic backgrounds, RIF was associated with a diet transition in which the intake of 11 dietary variables changed significantly. The main changes were increased sugar consumption and reduced consumption of starch. Despite an observed change of up to 10% in the intake of macronutrients, none of these changes were statistically significant. Observing changes in some nutritional factors and no changes in macronutrients indicates changes in food choices during Ramadan.

The diet adopted during Ramadan could explain a limited part of the changes in the metabolic profile following RIF. Consequently, a greater proportion of the changes in the metabolic profile is assumed to be due to the underlying mechanisms of fasting and other factors such as gut microbiome or lifestyle related changes (e.g., sleep, physical activity). This assumption is in agreement with the reported results in previous studies when adjusting for energy intake did not influence changes in blood pressure or body composition (22, 25). Moreover, the nutrient intakes changes did not explain much of the changes in SBP, DBP, weight, BMI, WC, HC, FM, F% and TBW.

### 4.2 Changes in metabolites, anthropometry and blood pressure

In LORANS, we investigated earlier changes in metabolites, anthropometry and blood pressure after RIF. We reported significant changes in 14 metabolite levels (normalized between 0 and 1) after RIF (26), namely lactate, acetate, glycoprotein acetyls, triglycerides in large high-density lipoprotein (HDL), triglycerides in intermediate-density lipoprotein (IDL), triglycerides in medium HDL, triglycerides in large low-density lipoprotein (LDL), triglycerides in HDL, tyrosine, pyruvate, acetone, triglycerides in LDL, phospholipids in very small very-low-density lipoprotein (VLDL), triglycerides in very small VLDL (*β* = −0.31, *P* = <0.001; −0.22, <0.001; −0.07, 0.006; −0.06, 0.006; −0.06, 0.007; −0.08, 0.013; −0.05, 0.014; −0.07, 0.019; −0.1, 0.019; −0.09, 0.019; 0.10, 0.019; −0.05, 0.035; −0.05, 0.041; −0.05, 0.041). However, the current study found that changes in metabolites after RIF are sex-dependent. Moreover, in terms of SBP, DBP and anthropometric measurements, we observed a reduction (22, 25) after RIF in SBP (−7.3, <0.001), DBP (−3.4, <0.001), weight (−1.6, <0.001), body mass index (−0.60, <0.001), waist circumference (−1.95, 0.011), hip circumference (−2.86, <0.001), fat mass (−1.24, <0.001), fat percentage (−1.05, <0.001) and total body water (−1.45, <0.001).

### 4.3 Comparison to previous studies

Although LORANS is the first study to report the changes in diet during Ramadan in a multi-ethnic group, its results agreed with studies that recruited samples from one ethnic background. In LORANS, the caloric intake was the same before and during Ramadan. This finding is consistent with most previous studies (3, 14, 19, 20, 27). Similarly, the intake and percentage contribution of the three macronutrients (protein, fat and carbohydrates) during Ramadan was the same as before. Likewise, numerous studies observed no change in the protein (1, 14, 17, 19, 20, 27, 28), fat (1, 13, 14, 19, 28) and carbohydrates (3, 19, 20) intake. Still, some studies reported a significant increase or decrease in some of these macronutrients (3, 15, 18, 20). The observed inconsistency could potentially stem from the fact that these studies were done in various countries, given that diet is tightly linked to culture. Nevertheless, two of the former studies were conducted in the same country (Iran) and yet reported inconsistent changes in macronutrients.

In LORANS, the intakes of nutritional factors such as cholesterol, sugars and starch were investigated. In agreement with a former study by Lamri-Senhadji et al. (13), we did not observe changes in cholesterol intake. The increase in sugar consumption by more than 50% in LORANS is in accordance with all former studies that observed higher sugar intake (3, 14, 19). None of the previous studies investigated the change in starch consumption, so we could not directly compare the observed decrease in our research to other studies. However, some foods rich in starch were found to be consumed less during Ramadan in some former studies (14, 18).

Although some former studies (29, 30) had assessed the changes in metabolites and lipids after RIF, the direct comparison of observed metabolic changes in LORANS to previous studies was not possible due to the use of different metabolic profiling methods.

### 4.4 Explaining changes in the metabolic profile

It is well-established that sugar intake is negatively associated with HDL and positively with total triglycerides (31); however, the increased sugar consumption in this study was associated with a non-significant reduction in HDL and no change in total triglycerides. Also, this cannot be explained by the non-significant drop in total fat intake and the different types of fat (saturated fat, monounsaturated and polyunsaturated). This might be due to the limited statistical power of our study and the effect of change in sugar intake in a month might be smaller than what our study could have detected. Moreover, the change in ketone bodies was almost independent of the changes in daily food intake. This was expected as the increased abundance of ketone bodies is due to compensating for the absence of glucose intake for more than 8 h, regardless of quantity of glucose intake before starting fasting, through converting ketogenic amino acids and fatty acids into ketones (32).

### 4.5 Strengths and limitations

This study has several strengths. Usin a 3-day food diary, including one weekend day, is a rigorous method to observe the day-to-day variation in food intake across the different days of the week compared to other methods (33). Also, the food diary reduced the risk of recall bias compared to the 24-h dietary recalls, dietary history and food frequency questionnaires. Translating the food diaries into nutrient data using “Nutritics” may have reduced human error in calculating the nutrients. In addition, LORANS is the only study investigating the dietary change during Ramadan in a multi-ethnic sample, giving an overall picture of the changes rather than specific changes in a certain culture. Moreover, the study was community-based, which makes it generalizable. Furthermore, utilizing the collected metabolomics data alongside the dietary data emphasized the magnitude of fasting effect on the metabolic profile apart from the diet adopted during Ramadan.

Also, this study presents some limitations. Although using the 3-day food diary was optimal for this study, it may have discouraged some participants from providing the dietary data due to the time and effort required and led to a low response rate (38.5%). Still, the number of participants who completed the food diary makes it among the largest studies on this topic, especially studies in which a food diary was used to measure diet.

Having a period of 8–12 days between the end of Ramadan and the second visit may have diluted the effect on the metabolic profile. In turn, it may have weakened the explanation of the change in the metabolic profile by diet change. However, reporting a significant difference in 14 metabolites after Ramadan indicates that the effect is still detectable.

## 5 Concluding comments

RIF is associated with a quick transition in dietary habits. The main changes are the higher consumption of sugars and the lower consumption of starch. However, the changes in the metabolic profile, blood pressure, and anthropometry after RIF are not merely due to the diet adopted during Ramadan.

## Data availability statement

The raw data supporting the conclusions of this article will be made available by the authors, without undue reservation.

## Ethics statement

The studies involving humans were approved by the Imperial College Research Ethics Committee. The studies were conducted in accordance with the local legislation and institutional requirements. The participants provided their written informed consent to participate in this study.

## Author contributions

RA-J: Data curation, Formal analysis, Visualization, Writing – original draft. YW: Writing – original draft, Writing – review & editing. PE: Writing – review & editing. KT: Conceptualization, Supervision, Writing – review & editing. AD: Conceptualization, Methodology, Supervision, Writing – review & editing.
