# Supplementary material for: The dietary changes during Ramadan and their impact on anthropometry, blood pressure, and metabolic profile
Source: Front Nutr. 2024 Jun 10;11:1394673. doi: 10.3389/fnut.2024.1394673 (PMC11194389; doi:10.3389/fnut.2024.1394673)
Supplement: Supplementary file 1 [file Data_Sheet_1.zip › Supplementary Material 1.PDF]

Confidential *Study No.* [ ]

Imperial College London

Food Diary

Please complete:

Date of birth: [ ][ ] / [ ][ ] / [ ][ ][ ][ ]

Gender: **Male** [ ] **Female** [ ]

Introduction

As we all know, diet is changed during the month of Ramadan and we are interested to have complete information of your diet before Ramadan and during Ramadan. We would like you to keep this diary of everything you eat and drink for three days before the month of Ramadan and three days during the month of Ramadan. This is a very important part of the study and will add greatly to the information you have already given us.

Please note that one day out of the three should be a weekend day (Saturday or Sunday)

It is very important that you do not adjust what you eat and drink just because you are keeping a record. Please continue to eat whatever you would normally eat.

Please provide us with as much detail as you possibly can.

Here are the dates you should record what you eat and drink:

Before Ramadan		During Ramadan	
Week day		Week day	
Week day		Week day	
Weekend day		Weekend day	

Please bring your booklet back when you come for the next visit on

……../……..../2019

If you have any queries please contact Rami AL Jafar

email: r.al-jafar18@imperial.ac.uk

Mobile: 07506380670

Thank you

General Instructions

1. For each day that you complete the food diary please write down everything you eat and drink within that 24-hour period. For example if you are completing the diary for Monday you need to start recording from midnight on Sunday night until midnight on Monday.

   - Using the 24-hour clock please note the time that you have something to eat or drink. Please write down all the food and drink you consumed at that time, the amounts and a description.

   - Please remember to record all drinks including alcohol, hot drinks, and items such as crisps, nuts, chocolates and sweets.

   - If you prepare a recipe, please write it in the recipe box provided at the end of each day.

2. Please try to record everything at the time of eating, not from memory at the end of the day.

3. Please read pages 4-9 for help in describing the foods and drinks you have eaten. Pages 10-16 include a range of photographs and page 17 shows an example of part of a completed diary.

4. Give **brand and full name** of products from packaging. Many commercial foods have **weights** printed on them, so please use these to show how much you ate.

5. Please answer the questions at the back of the diary, **after** you have completed for three days before Ramadan (pages 18 - 34) and three days during Ramadan (pages 35-51).

Detailed Instructions

The following section is a list of popular foods and drinks. Next to each item is the sort of thing we need to know so that we can tell what it is made of and how much you had. This list cannot cover all foods and drinks, so if anything that you have eaten is missing try to relate it to a similar item. Please give as much detail as you can. As an example, see page 17.

Please try to state what sort of oil or fat was used for baking, frying etc.

State clearly whether spread was used on crackers and biscuits as well as on bread, rolls, toast and in sandwiches.

Food/Drink	Description & Preparation	Brand	Amount
Homemade dishes	Describe as fully as possible, include name of dish; give recipe or ingredients, including amounts if known		Tablespoons<br>One of the suitable photos
Ready-made meals	Give name of dish as described on pack with brand, describe main ingredients and enclose label e.g. beef lasagne, deep pan pizza, fish pie etc.	Sainsbury's, Tesco, Morrisons, Asda	Weight from packet including proportion of pack eaten (all or half?) Tablespoons; one of the suitable photos.
Meals eaten away from home or take-away meals	Please describe all dishes and give main ingredients e.g. lamb tikka masala and pilau rice, other Indian and oriental dishes, fish and chips, burgers, pizza etc.	McDonalds, Pret a Manger, Costa Coffee, Greggs, pizza Express, Nando's	Proportion of takeaway or restaurant carton<br>Describe meal size and dimensions where appropriate.<br>Tablespoons; one of the suitable photos

BEVERAGE			
Fruit juice<br>*Fruit drinks*<br>*Soft drinks*	Without added sugar<br>With added sugar<br>Brand name, regular or diet or low calorie	Oasis, Capri, Ribena, Robinsons no added sugar, Pepsi Max	Glasses, cartons, cans or bottles with volume
Coffee	Instant or ground; decaffeinated or caffeinated; with milk or sugar		Cups or mugs<br>Volume if available
Tea	Tea leaves or tea bag, with milk or sugar. If instant: black or white, sweetened or not		Cups or mugs<br>Volume if available

Milk based or hot chocolate type drinks	Name of type of drink; regular, reduced fat or low sugar. Type of milk used		Cups or mugs<br>Volume if available
Water	Tap, bottled or filtered		Glass, tumbler; volume
**BISCUITS/ CRACKERS**			
Sweet biscuits	Brand and full product name plus description e.g. sandwich, wafer, chocolate half-coated, full-coated, cream-filled<br>Ingredients if homemade	Penguin, Hub Nob, Fox's, Maryland	Number of biscuits and size
Crackers, crisp bread, savoury biscuits	Brand and full product name plus description	Carr's water biscuits, Original Ryvita, Jacob's Choicegrain	Number of crackers and size
**BREAD**			
Bread	White, brown, granary, wholemeal, containing seeds, ciabatta, focaccia, french type, baguette.<br>Was the loaf pre-sliced or hand-cut?		Size of loaf: large or small<br>Thickness of slice<br>Number of slices
Rolls or buns	Describe rolls: crusty, soft, baps, petit pain, finger		Size of rolls and number
Sandwiches	Remember to describe type and amount of spread and filling		Size of rolls or slices of bread
**BREAKFAST CEREALS**			
Breakfast cereal	Brand and full name<br>Remember to describe milk and sugar added separately	Jordan's<br>Natural Muesli,<br>Sainsbury's Malties	Photo 1<br>Tablespoons<br>Milk on cereal: large, medium or small amount
Porridge or Ready Brek	Porridge oats or Ready Brek<br>Type of milk used to make it or was water use?<br>Remember to describe milk and sugar added separately		Photo 1<br>Amount of ingredients
Bran: wheatbran, wheatgerm oatgerm and bran	Added separately to breakfast cereal or mixed with other foods such as porridge. Please describe type and brand.		Dessertspoons or tablespoons

BUTTER, MARGARINES, FATS & OILS			
Butter, spreads or margarines	Please give specific brand, full names as described on packaging plus the percentage (%) fat if known	Flora Light, Clover, Bertolli, Lurpack Spreadable	Photo 18 for spread bread or rolls<br>For crackers and biscuits describe thickness of spread
Oils	Describe type of oil used in cooking or dressings e.g. corn, olive, sunflower		Tablespoons
**CAKES**			
Cakes, scones and sweet buns, pies and pastries	Homemade – describe ingredients and recipe<br>Commercial – give brand and product name with description<br>Does cake contain filling e.g. whipped cream, butter icing or have a coating or covering?<br>Are pies made with pastry top and bottom?		Proportion of whole cake or pie<br>Size of slice or individual cake<br>Photo 15 and 16 for cake<br>Photo 3 for pies or flans
	Are scones or cakes spread with butter, margarine and/or jam?		How many whole scones or halves?
**CHEESE**			
Hard cheese (includes Brie, Danish Blue etc.)	Specify type	Cheddar, Wensleydale, Brie	Photo 2 (amount eaten is equal to the slice OR the chunk OR the grated cheese)<br>Number and size of slices or chunks
Philadelphia type soft cheese or cheese spread	Regular or reduced fat cheese specify brand and fat content		Thick or thin spread teaspoons
**DESSERT/PUDDINGS**			
Puddings	Describe type and ingredients e.g. apple crumble, raspberry cheesecake with biscuit base, dairy cream trifle with banana<br>Served with custard, ice cream, cream or yogurt? (see milk).		Photo 17; tablespoons<br>Size of slice; weight of carton for commercial item<br>Photo 3 for pies or flans
**EGGS**			
Eggs and dishes	Boiled, poached, fried, scrambled, omelette plus topping or other ingredients<br>Was fat or oil used in cooking?<br>Give type of fat or oil used		Size of eggs<br>Number of eggs consumed

**FISH**			
Fish and fish dishes	Type of fish; fresh, frozen or canned, cooking method; from fish and chip shop, homemade or commercial; battered or breadcrumber		Weight (with or without bones/skin?);<br>size of whole or piece of fish<br>Photo 6
**FRUIT**			
Fruit	Type of fruit; fresh (was skin eaten or not?), frozen, dried; stewed with or without sugar<br>Canned in syrup or juice		Number of whole fruits<br>Tablespoons; weight (with or without skin)<br>Weight of can
**MEAT**			
Red meat	Type Cut from joint or pre-sliced		Weight; number and size or thickness of slices<br>Photo 4
Sausages	Type, cooking method		Number and size
Chops and steaks	Type and cut, cooking method<br>Was the fat eaten?		Number and size<br>Weight (raw or cooked)
Meat dishes	Recipe or brand and product name<br>with ingredients		Photo 5, 19, or 20<br>Tablespoons; pack weight
**MILK/DAIRY**			
Milk	Whole, semi-skimmed or skimmed; percentage (%) fat if known<br>Pasteurised, UHT or sterilised		Tablespoons<br>Volume in fl.oz. or ml.
Powdered milk	Dried skimmed milk or with added vegetable fat		Teaspoons; volume of made up milk
Coffee or tea creamer or whitener	Brand and product name<br>Please state if powder or liquid	Coffeemate	Teaspoons<br>Individual cartons or sachets
cream	Single, whipping or double; dairy or non-dairy; regular or reduced fat<br>Liquid, whipped or aerosol		Tablespoons<br>Volume
Yogurt and fromage frais	Brand and specific product name or description, fat content as on carton	Muller Corner, Activia 0%, Weight Watchers, Tesco	Tablespoons; size of carton (g. or ml.)
Ice cream	Brand and product name; regular, reduced fat or made with cream		Scoops<br>Tablespoons
Non-dairy milk	Soya, oat or rice milk; brand; product description; fortified with calcium; sweetened?		Tablespoons<br>Volume

**PASTA**			
Pasta and spaghetti incl. filled pasta	Dried or fresh pasta; white or wholemeal; describe type e.g. fusilli or tagliatelle<br>Filled pasta e.g. Tortelloni with spinach and ricotta		Weight (raw or cooked)<br>Photo 9<br>Proportion of packet weight
Pasta dishes	Lasagne, cannelloni or pasta bakes; give recipe and ingredients for homemade; brand, product name and description for commercial		Photo 20<br>Packet weight
Pasta sauce	Describe sauce type and ingredients		Tablespoons<br>Volume or weight of commercial product
**RICE**			
Rice	White or brown, long grain or basmati		Photo 8<br>Weight (raw or cooked)
Rice dishes	Give recipe and ingredients for homemade; brand, product name and description for commercial		Photo 8; tablespoons<br>Packet weight and proportion eaten
**SAUCES & SOUPS**			
Sauces and ketchups including dips	Describe brand and product name or recipe and ingredients		Tablespoon or teaspoons<br>Volume or weight of commercial product
Soups	Describe type and ingredients<br>Is soup homemade, canned, condensed, dried packet, instant, fresh/carton or low calorie?		Bowls, cups or mugs<br>Volume in fl.oz. or ml.<br>Weight of can and proportion eaten
Gravy	Describe brand and product name or recipe and ingredients<br>Made with cornflour, bisto powder, granules; with or without added meat juices, stock or vegetable juice		Tablespoons<br>Volume in ml. or fl.oz.
Dressings	Type and ingredients; brand and product name; regular, reduced fat or fat free		Tablespoons or teaspoons
Mayonnaise	Regular or reduced fat		Tablespoons etc.
**SAVOURY DISHES**			
Pies, flans and quiches<br>*Pizza*<br>*Pancakes*<br>*Sausage rolls*<br>*Filled tortillas or burritos*	Describe dish and ingredients, brand and product name		Product weight and proportion eaten<br>Number of slices or individual items eaten

SAVOURY SNACKS			
Crisps and snacks<br>*Nuts*	Brand name and description<br>Type; fresh or roasted; salted or unsalted		Weight of packet<br>Number of items eaten
**SPREADS & CONDIMENTS**			
Jams, other preserves and spreads	Brand name and type of spread<br>Jam, honey, marmalade<br>Peanut butter, other nut butters<br>Chocolate spread<br>Marmite and savoury spread		Thin, medium or thick spread
Salt, pepper, mustard	Describe type		Sprinkle; teaspoons
**SUGARS & CONFECTIONERY**			
Sweets and chocolate	Describe type and brand		Weight; number of pieces, whole bars or individual sweets
Sugars and sweeteners	Type of sugar<br>Brand and type of sweetener		Teaspoons<br>Tablets or spoons
**VEGETABLES (including herbs)**			
Vegetables and salad including lentils, beans and baked beans	Type of vegetables; fresh, frozen or canned; cooking method or raw<br>If roasted was fat added?<br>Was butter, sauce or dressing added?		Photo 12,13 or 14<br>Number of whole vegetables<br>Tablespoons
Vegetable dishes including dishes with potato, beans, lentils or pulses	Recipe or brand and product name with ingredients		Photo 5 or 20<br>Weight of commercial dish
Potatoes	Boiled; roasted with or without fat; fried; sautéed; mashed with or without added fat or milk		Photo 10 or 11
Chips	Homemade; commercial e.g. oven chips; takeaway<br>Size and cut of chip		Photo 7
Herbs and spices	Fresh or dried		Teaspoons or other spoons; leaves; sprigs
**VEGETARIAN**			
Vegetarian products and dishes with Quorn, soya or TVP or tofu	Describe dish or product and ingredients, brand and product name e.g. Quorn sausages, Vegetable stir-fry with tofu		Weight from packaging<br>Number of items<br>Number of slices of meat substitute<br>Photo 5<br>Tablespoons

Please choose an appropriate photo to indicate the portion size you have eaten. To help you make this choice, there are some notes below the photos. Write down the picture number and size nearest to your own helping e.g. 2a, 3b or 1c.
The large white circle in the background shows the actual size of the 10” dinner plates used in the photos. Items such as the cake are photographed on a 7” tea plate.
Refer to the detailed instructions on pages 4 - 9 where * is indicated.

10” Plate in pictures

1 a	1b	1c
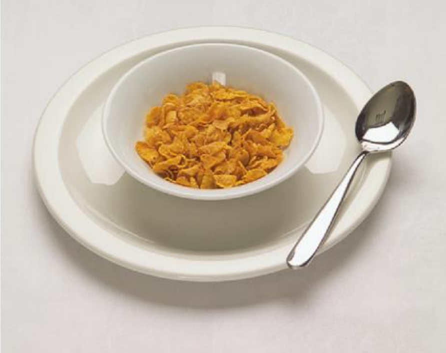	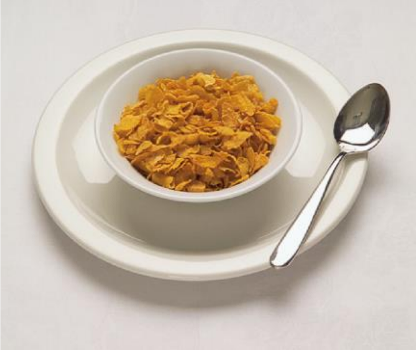	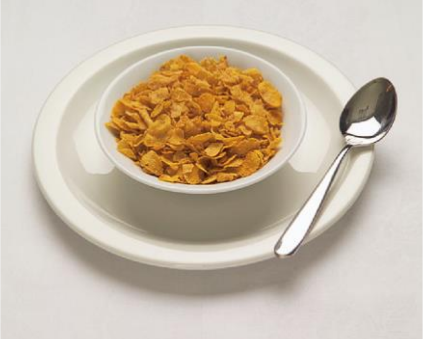
Suitable for - Corn flakes and other breakfast cereals		

2 a	2b	2c
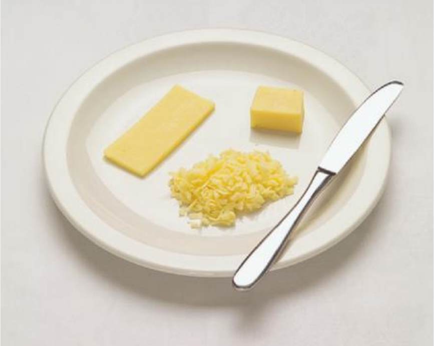	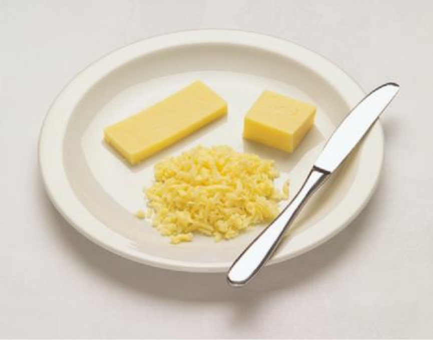	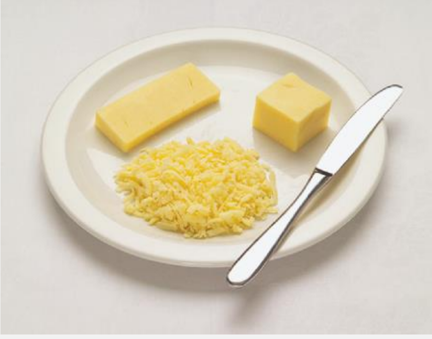
Suitable for - Cheese<br>Not for - Butter, margarines and spreads (see photo 18)		
PLEASE NOTE: *When choosing one of the photos above, the amount you eat is equal to either the slice* ***OR*** *the chunk* ***OR*** *the grated cheese on one plate*		

3 a	3b	3c
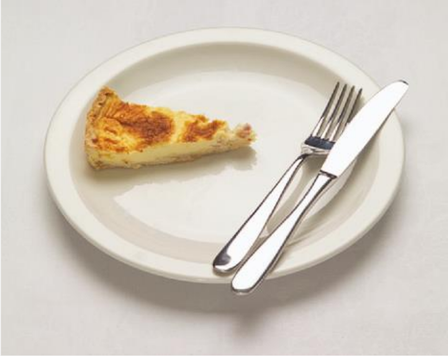	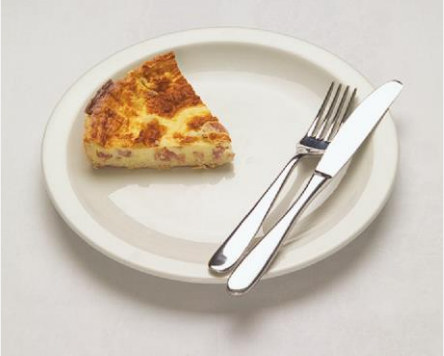	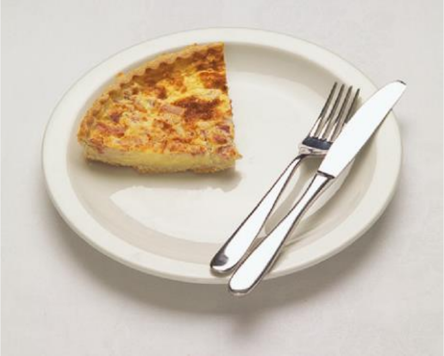

Suitable for - Quiches, flans, savoury or sweet pies and pizza
Not for - Cakes (see photos 15 and 16)

4 a	4b	4c
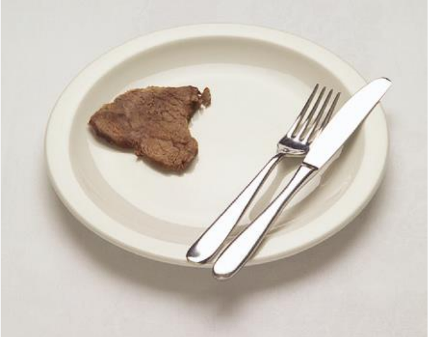	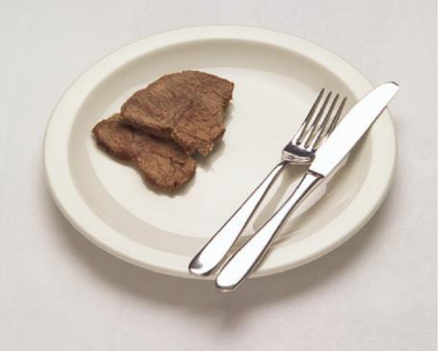	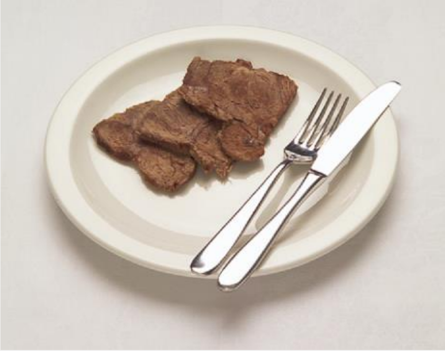

Suitable for - Hot or cold sliced meats, e.g. roast meat, ham or gammon
Not for - Chops, steaks or bacon rashers *

5 a	5b	5c
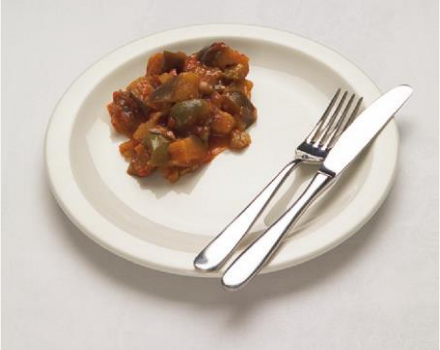	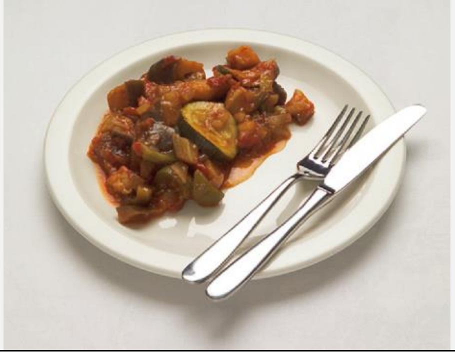	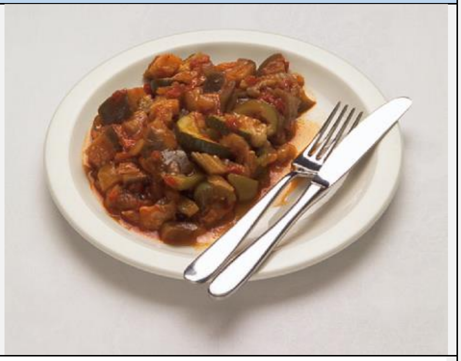

Suitable for - Vegetable stews or meat stews and casseroles WITH vegetables, also bolognaise sauce
Not for - Meat stews WITHOUT vegetables (see photo 19)

6 a	6b	6c
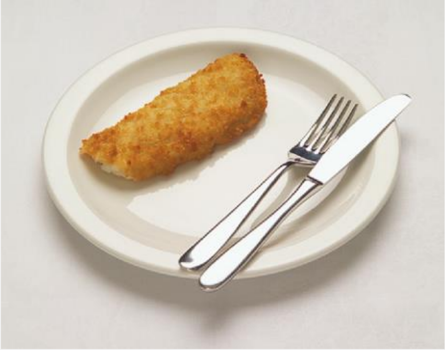	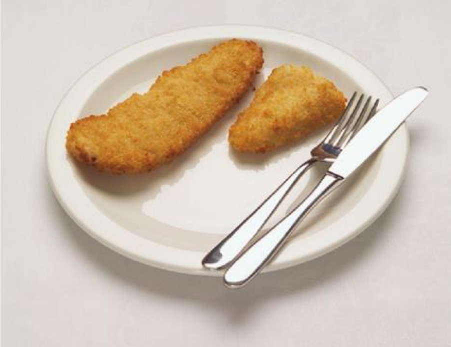	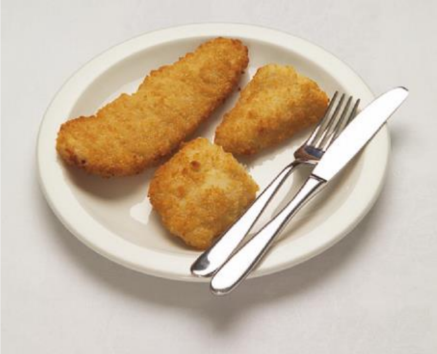
Suitable for - Fish including fish in breadcrumbs or batter<br>Not for - Chops or steaks *		

7 a	7b	7c
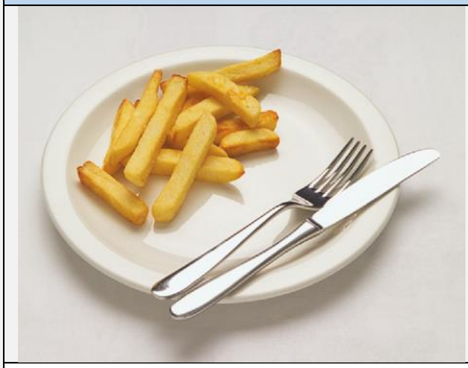	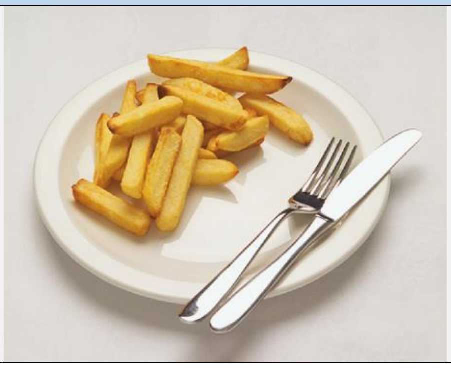	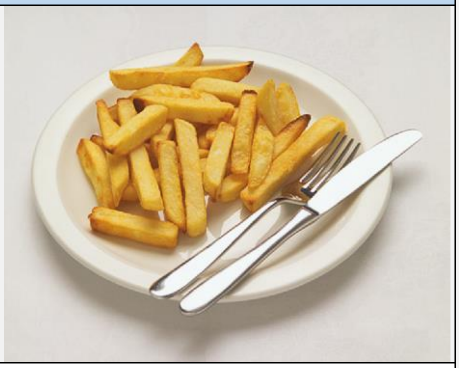
Suitable for - Chips only		

8 a	8b	8c
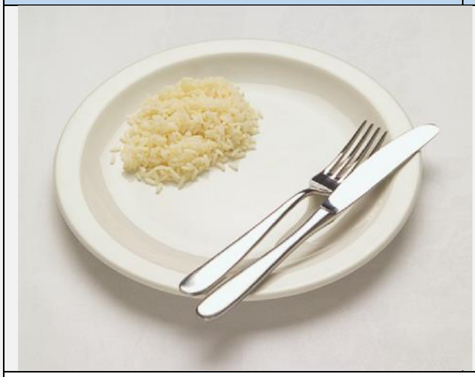	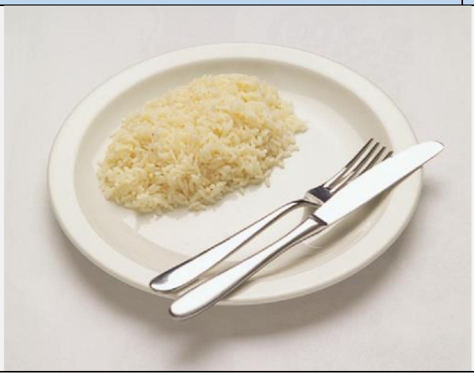	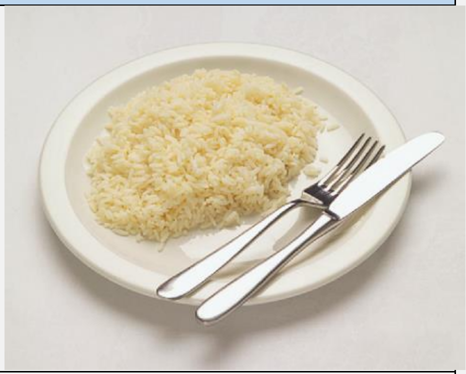
Suitable for - Boiled rice and rice dishes		

9 a	9b	9c
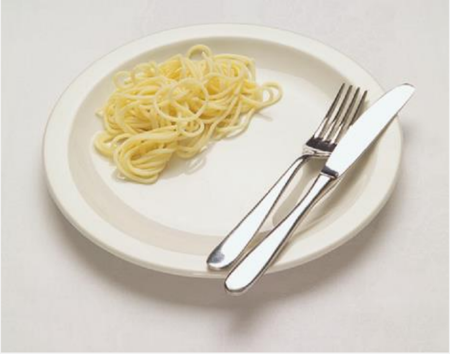	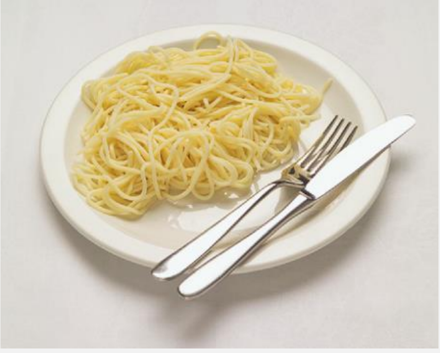	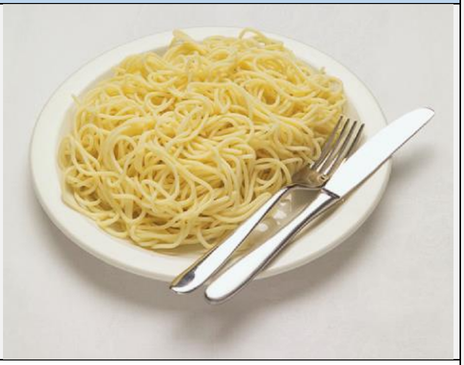
Suitable for - Boiled spaghetti, other boiled pastas and noodles plus pasta and noodle dishes		

10 a	10b	10c
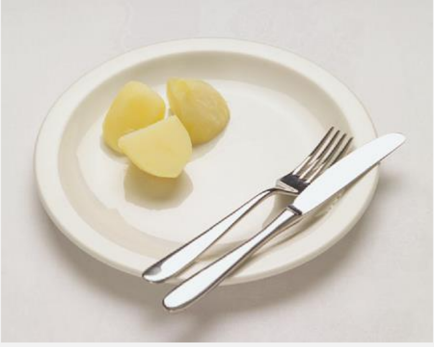	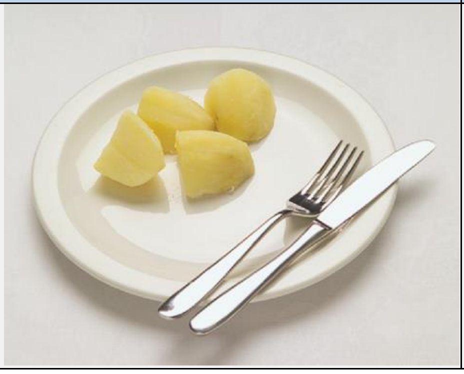	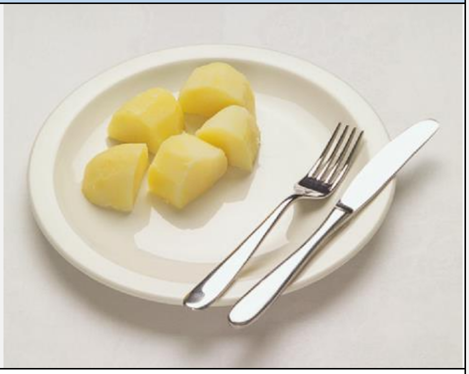
Suitable for – Boiled or roast potato		

11 a	11b	11c
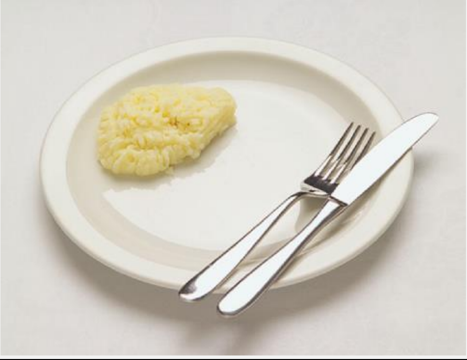	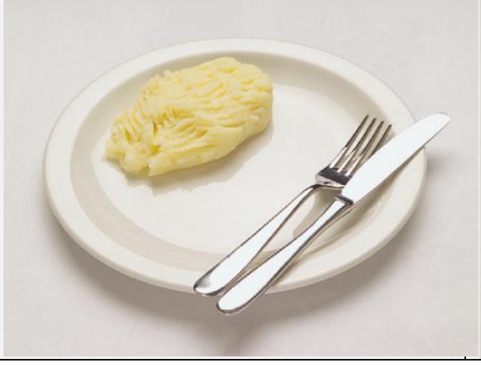	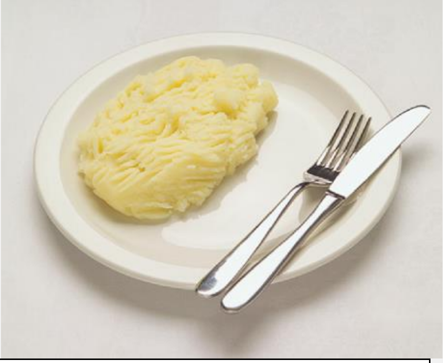
Suitable for – Mashed potato		

12 a	12b	12c
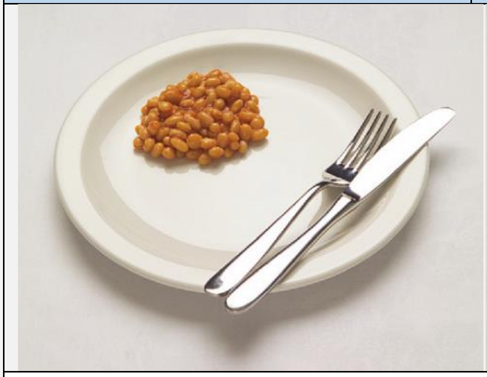	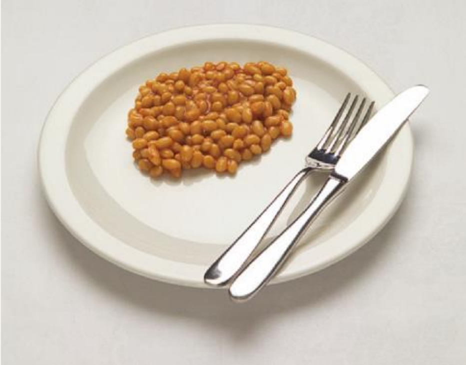	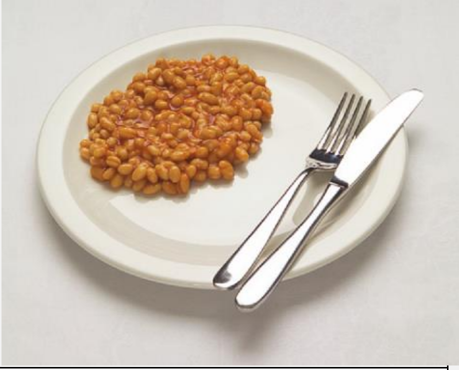
Suitable for – Baked beans and peas		

13 a	13b	13c
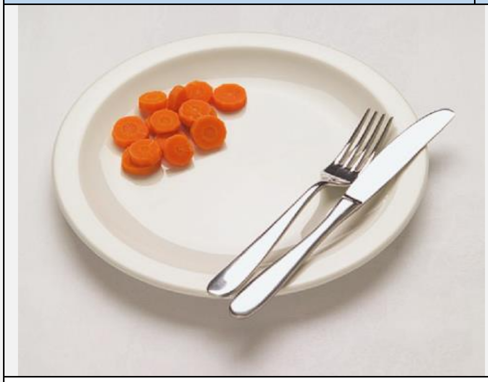	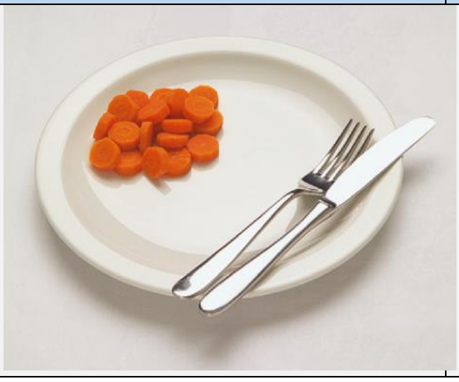	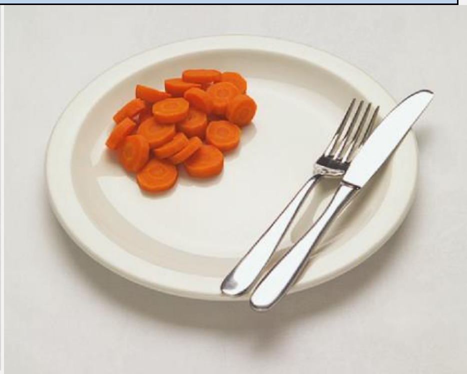
Suitable for – Carrots and other similar vegetables		

14 a	14b	14c
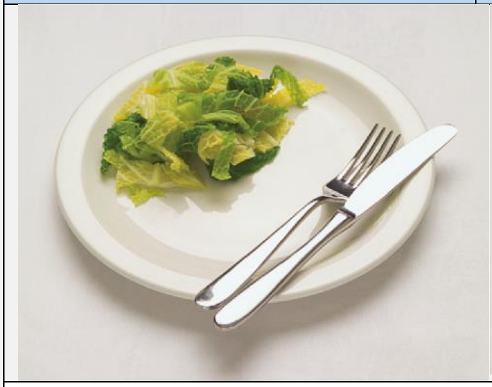	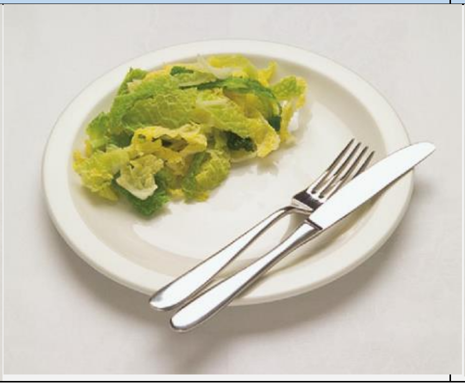	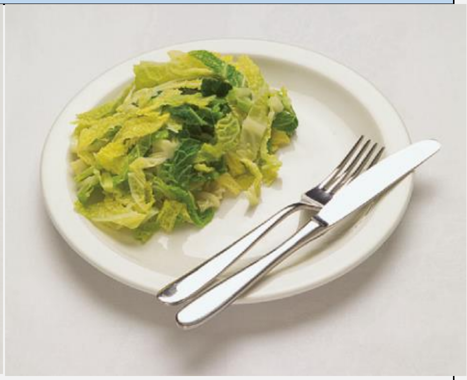
Suitable for – Cabbage, other leafy vegetables and salads<br>Not for – Peas (see photo 12)		

15 a	15b	15c
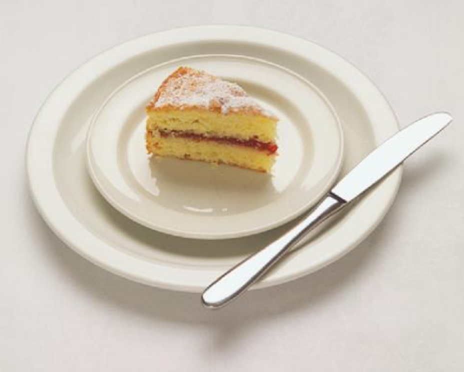	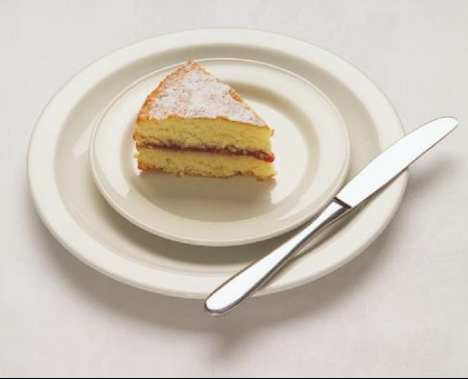	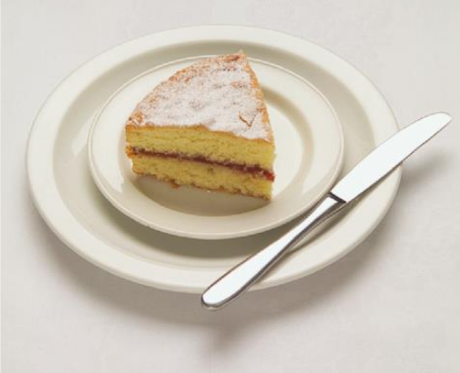

Suitable for – Sponge cake and other similar cakes
Not for – Quiches, flans and sweet or savoury pies (see photo 3)

16 a	16b	16c
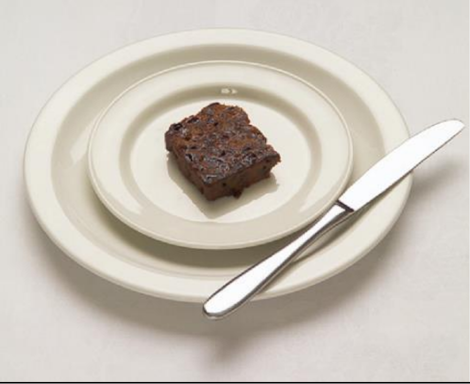	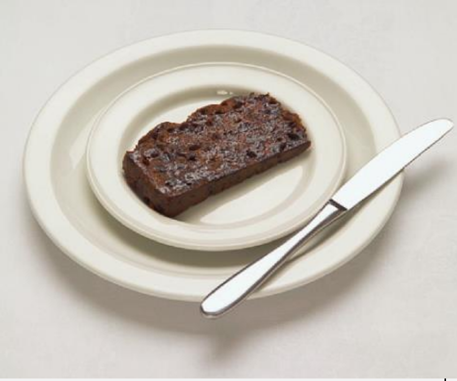	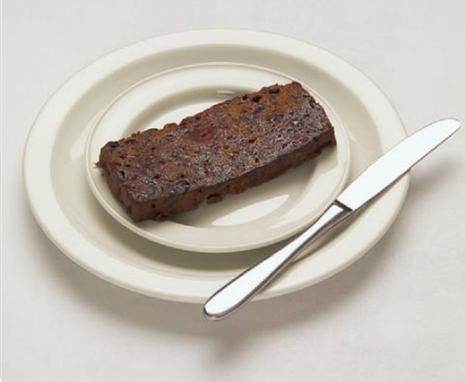

Suitable for - Fruit cake and other cake types with same shape
Not for - Meat (see photo 4) *

17 a	17b	17c
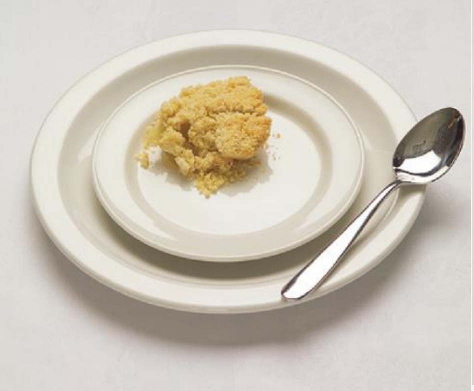	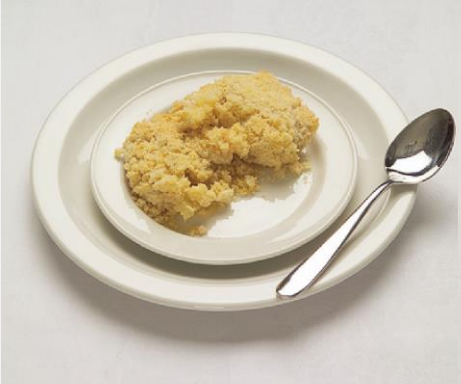	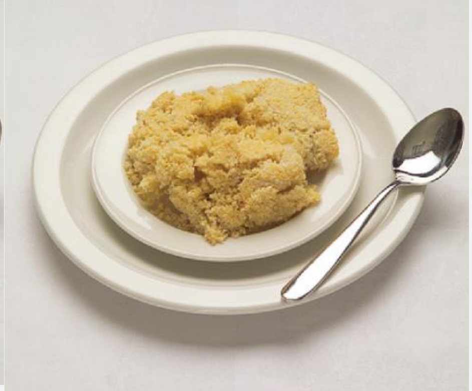

Suitable for - Fruit crumble and other puddings and desserts
Not for - Puddings WITH custard, sauce, yoghurt or ice cream combined *

18 a	18b	18c
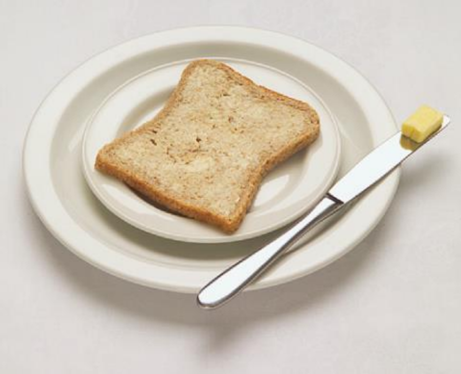	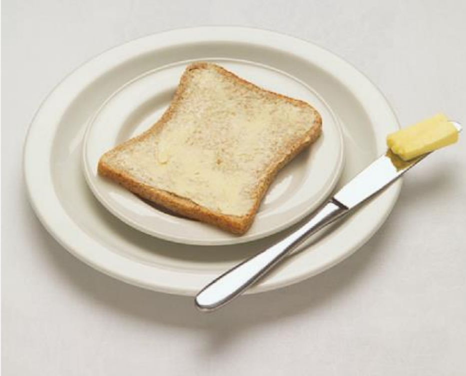	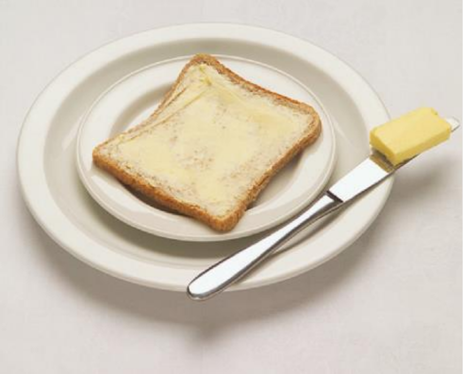

Suitable for – Butter, margarines and spreads on bread only

19 a	19b	19c
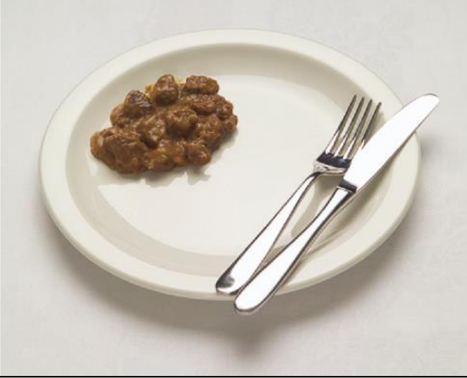	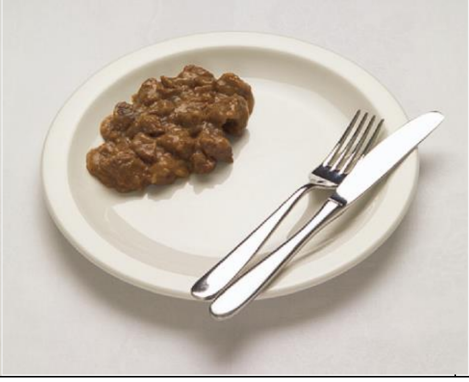	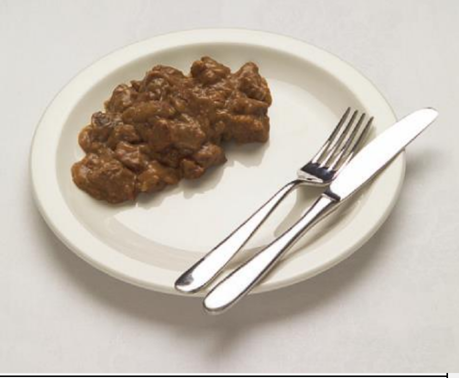

Suitable for – Meat or minced meat stews WITHOUT vegetables
Not for – Meat stews WITH vegetables (see photo 5)

20 a	20b	20c
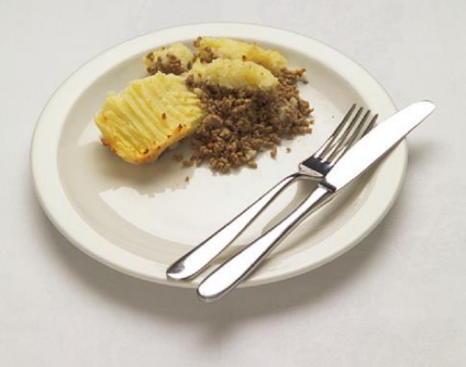	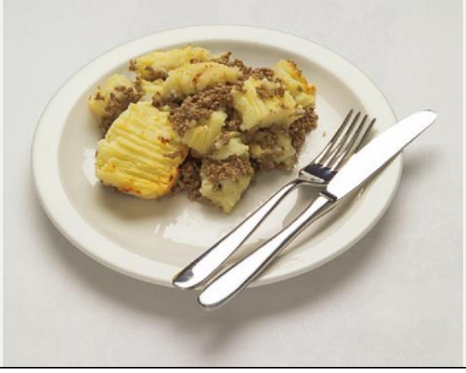	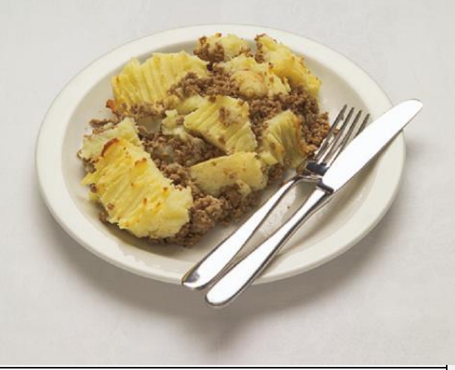

Suitable for – Shepherd’s pie and similar dishes or lasagne

Start at 0:00 hours and end at 23:59. Please record using the 24-hours clock.

EXAMPLE

Please refer to the detailed instructions on pages 4 - 16

DATE: 07/03/2019		DAY OF THE WEEK: Wednesday		
Time	Food/drink	Description and preparation	Brand	Amount
05:00	Tea			
	Water	Tap water		1 small glass
08:00	Yoghurt			
13:00	Beef casserole	Beef casserole (onion and carrots)	Bought at	Photo 5 b
	Potato	Mashed potato	Staff	2 scoops
	Vegetable	Boiled cabbage	canteen	Photo 14 a
	Dessert	Rhubarb crumble		Photo 17 b
		Custard		2 small ladles
	Tea	Tea bag		1 plastic cup
		Semi skimmed milk		1 tbsp
17:00	Apple	Braeburn – ate skin		1 small fruit
	Chocolate	Milk chocolate	Cadbury's	1 X 49g bar
	Tea	Tea bag		1 mug
		Semi skimmed milk		2 tbsp
20:00	Chicken and	Skinless and boneless chicken breast		300 g raw ate
	vegetable	Vegetable oil		1 tsp ½
	Stir-fry	Black bean stir-fry sauce	Sharwood's	195g jar of this
		Button mushrooms		4 oz recipe
	Rice	With rice boil in the bag	Uncle Ben's	½ Bag
	Water	Tap water		1 small glass

START OF DAY NO. 1 (before Ramadan)

DATE:	DAY OF THE WEEK:

Time	Food/drink	Description and preparation	Brand	Amount

Time	Food/drink	Description and preparation	Brand	Amount

Time	Food/drink	Description and preparation	Brand	Amount

**Have you forgotten anything?**<br>Please write below any other items that you had to eat and drink today that have **NOT** already been written. Please remember to write the time at which you ate/drank them				
Food/Drink	Description and preparation	Brand	Amount	Time
Chocolate				
Toffees, sweets				
Crisps, peanuts				
Other snacks				
Other cold				
Drinks				
Tea, coffee				
Other hot drinks				
Ice cream				
Anything else?				

Space to write in the recipe or ingredients of any home-made dishes, take-away meals etc. that you have mentioned but not described previously. Where applicable, please list amounts of ingredients and brand names. **Please indicate the amount or proportion actually consumed by yourself.**

**END OF DAY No. 1**

START OF DAY NO. 2 (<u>before Ramadan)</u>

DATE:	DAY OF THE WEEK:

Time	Food/drink	Description and preparation	Brand	Amount

Time	Food/drink	Description and preparation	Brand	Amount

Time	Food/drink	Description and preparation	Brand	Amount

Have you forgotten anything?

Please write below any other items that you had to eat and drink today that have **NOT** already been written. Please remember to write the time at which you ate/drank them

Food/Drink	Description and preparation	Brand	Amount	Time
Chocolate				
Toffees, sweets				
Crisps, peanuts				
Other snacks				
Other cold				
Drinks				
Tea, coffee				
Other hot drinks				
Ice cream				
Anything else?				

Space to write in the recipe or ingredients of any home-made dishes, take-away meals etc. that you have mentioned but not described previously. Where applicable, please list amounts of ingredients and brand names. **Please indicate the amount or proportion actually consumed by yourself.**

END OF DAY No. 2

START OF DAY NO. 3 (<u>before Ramadan)</u>

DATE:	DAY OF THE WEEK:

Time	Food/drink	Description and preparation	Brand	Amount

Time	Food/drink	Description and preparation	Brand	Amount

Time	Food/drink	Description and preparation	Brand	Amount

**Have you forgotten anything?**<br>Please write below any other items that you had to eat and drink today that have **NOT** already been written. Please remember to write the time at which you ate/drank them				
Food/Drink	Description and preparation	Brand	Amount	Time
Chocolate				
Toffees, sweets				
Crisps, peanuts				
Other snacks				
Other cold				
Drinks				
Tea, coffee				
Other hot drinks				
Ice cream				
Anything else?				

Space to write in the recipe or ingredients of any home-made dishes, take-away meals etc. that you have mentioned but not described previously. Where applicable, please list amounts of ingredients and brand names. **Please indicate the amount or proportion actually consumed by yourself.**

**END OF DAY No. 3**

GENERAL QUESTIONS ABOUT YOUR FOOD/DRINK DURING LAST WEEK

1. *Which type of milk did you most often use last week?* ***Select one only.***

☐ Whole/full cream
☐ Semi-skimmed
☐ Skimmed/fat free
☐ Soya
☐ Other:
☐ No milk used

Do you know the fat percentage (%) of your milk?: ☐

Was this milk: ☐ pasteurized? ☐ UHT? ☐ Sterilizer? ☐ dried?

2. *How much milk did you usually have in tea, coffee and on your cereal?*

Tea:	☐ A lot	☐ Average	☐ Hardly any	☐ No milk used
Coffee:	☐ A lot	☐ Average	☐ Hardly any	☐ No milk used
cereal:	☐ A lot	☐ Average	☐ Hardly any	☐ No milk used

3. *Did you drink decaffeinated tea or coffee?*

Tea:	☐ Always	☐ Sometimes	☐ Never
Coffee:	☐ Always	☐ Sometimes	☐ Never

4. *Which types of fat did you use last week for baking, frying, spreading and on salads?* **If you are not sure which category to indicate, check packaging for the exact name, fat content and brand and fill in this information**

Type of fat, spread or margarine	Brand and name or product	Spreading	Frying	Baking	Salads
Butter					
Spreadable butter					
Diary spread (e.g. I can't believe it's not butter)					
Polyunsaturated spread (sunflower, soya or vegan)					
Low fat spread (less than 60% fat)					

Type of fat, spread or margarine	Brand and name or product	Spreading	Frying	Baking	Salads
Olive oil based spread					
Other soft margarine or spread - 1					
Other soft margarine or spread - 2					
Hard margarine					
Vegetable oil - 1					
Vegetable oil - 2					
Lard					
White vegetable fat					
Dripping or animal fat					
Other					

5. *Which type of bread did you eat most often last week?*
Select one only.

- ☐ White
- ☐ Soft grain
- ☐ Granary
- ☐ Brown
- ☐ Wholemeal
- ☐ wheatgerm

Other: ☐

6. *Did you eat butter, margarine or spread last week?*
Please tick boxes below to show whether you ate it on toast, bread, sandwiches, in rolls or on crackers:

	Toast	Bread	Sandwiches	Rolls	Crackers
Always					
Sometimes					
Never					
Don't know					

7. *How thickly did you spread butter, margarine etc. on bread or crackers?*

- ☐ Thick
- ☐ Medium
- ☐ Thin
- ☐ None

8. *If you ate grilled, fried, barbecued or roast meat last week, how well cooked was it?*
Please tick the boxes.

	Red meat: e.g. Beef, lamb	Poultry
Well done or dark brown		
Medium		
Lightly cooked or rare		
Did not eat meats cooked by these methods		
Did not eat these meats		

9. *If you ate red meat last week, what did you do with the visible fat?*
Please note that red meat includes beef, lamb, etc.

☐ Ate all of the fat ☐ Ate most of the fat
☐ Ate some of the fat ☐ Ate as little as possible
☐ Did not eat meat ☐ No fat eaten

10. *If you ate poultry last week, did you eat the skin?* ***Please note that poultry includes chicken, duck, goose and game birds.***

☐ Yes ☐ No
☐ Sometimes ☐ Did not eat poultry

11. *If you had gravy last week, were the meat juices, pan residues or dripping put into the gravy?*

☐ Yes ☐ No ☐ Sometimes
☐ Don't know ☐ Did not eat gravy

12. *Was salt usually added to your food during cooking last week?*

☐ Yes ☐ No ☐ Don't know

Did you usually add salt to your food at the table last week?

☐ Yes ☐ No ☐ Don't know

Did you regularly use a salt substitute (e.g. LoSalt) last week?

☐ Yes ☐ No ☐ Don't know

13. *Did you eat the skin on fruit?* ***Please tick boxes.***

	Apple	Pear
Skin eaten		
Skin not eaten		
Fruit not eaten		

14. *Please name any vitamins, minerals or other food supplements taken on each day of last week.* ***Please write down all the details from each packet/container or enclose label(s). Give the number of tablets taken on each day.***

Example

Name	Strength	capsule	Tablet tsp.	Day 1	Day 2	Day 3
Boots	High strength vitamin C	1000 mg	Tablet	1	1	2

15. *Which types of water did you consume last week?*
Please give information for both HOT and COLD drinks.

Water type	Hot drink	Cold drink
Tap water (unfiltered)		
Filtered water – hard water filter		
Filtered water – other		
Bottled water – *brand:*		
Other water – *brand:*		

16. *Were any of the following foods which you ate last week produced **organically** (without pesticides)? **Please tick the necessary box(es).***

- ☐ Vegetables, homegrown
- ☐ vegetables, purchased
- ☐ Fruits, homegrown
- ☐ Fruit, purchased
- ☐ Milk and diary products
- ☐ cereal or cereal products, bread
- ☐ Meat
- ☐ No organic foods eaten

**This space has been left for you to tell us about anything else which you feel is important about your food/drink intake in last week.**

Please bring the diary back with you to the clinic for your health screening appointment.

Thank you very much for your help

in completing such a detailed record

START OF DAY NO. 1 (<u>during Ramadan</u>)

DATE:			DAY OF THE WEEK:	
Time	Food/drink	Description and preparation	Brand	Amount

Time	Food/drink	Description and preparation	Brand	Amount

Time	Food/drink	Description and preparation	Brand	Amount

Have you forgotten anything?<br>Please write below any other items that you had to eat and drink today that have **NOT** already been written. Please remember to write the time at which you ate/drank them				
Food/Drink	Description and preparation	Brand	Amount	Time
Chocolate				
Toffees, sweets				
Crisps, peanuts				
Other snacks				
Other cold				
Drinks				
Tea, coffee				
Other hot drinks				
Ice cream				
Anything else?				

Space to write in the recipe or ingredients of any home-made dishes, take-away meals etc. that you have mentioned but not described previously. Where applicable, please list amounts of ingredients and brand names. **Please indicate the amount or proportion actually consumed by yourself.**

END OF DAY No. 1

START OF DAY NO. 2 (during Ramadan)

DATE:		DAY OF THE WEEK:		
Time	Food/drink	Description and preparation	Brand	Amount

Time	Food/drink	Description and preparation	Brand	Amount

Time	Food/drink	Description and preparation	Brand	Amount

Have you forgotten anything? Please write below any other items that you had to eat and drink today that have **NOT** already been written. Please remember to write the time at which you ate/drank them				
Food/Drink	Description and preparation	Brand	Amount	Time
Chocolate				
Toffees, sweets				
Crisps, peanuts				
Other snacks				
Other cold				
Drinks				
Tea, coffee				
Other hot drinks				
Ice cream				
Anything else?				

Space to write in the recipe or ingredients of any home-made dishes, take-away meals etc. that you have mentioned but not described previously. Where applicable, please list amounts of ingredients and brand names. **Please indicate the amount or proportion actually consumed by yourself.**

**END OF DAY No. 2**

START OF DAY NO. 3 (<u>during Ramadan)</u>

DATE:	DAY OF THE WEEK:

Time	Food/drink	Description and preparation	Brand	Amount

Time	Food/drink	Description and preparation	Brand	Amount

Time	Food/drink	Description and preparation	Brand	Amount

Have you forgotten anything?

Please write below any other items that you had to eat and drink today that have **NOT** already been written. Please remember to write the time at which you ate/drank them

Food/Drink	Description and preparation	Brand	Amount	Time
Chocolate				
Toffees, sweets				
Crisps, peanuts				
Other snacks				
Other cold				
Drinks				
Tea, coffee				
Other hot drinks				
Ice cream				
Anything else?				

Space to write in the recipe or ingredients of any home-made dishes, take-away meals etc. that you have mentioned but not described previously. Where applicable, please list amounts of ingredients and brand names. **Please indicate the amount or proportion actually consumed by yourself.**

**END OF DAY No. 3**

GENERAL QUESTIONS ABOUT YOUR FOOD/DRINK DURING LAST WEEK

1. *Which type of milk did you most often use last week?* ***Select one only.***

☐ Whole/full cream ☐ Soya

☐ Semi-skimmed ☐ Other:

☐ Skimmed/fat free ☐ No milk used

Do you know the fat percentage (%) of your milk?: ☐

Was this milk: ☐ pasteurized? ☐ UHT? ☐ Sterilizer? ☐ dried?

2. *How much milk did you usually have in tea, coffee and on your cereal?*

Tea:	☐ A lot	☐ Average	☐ Hardly any	☐ No milk used
Coffee:	☐ A lot	☐ Average	☐ Hardly any	☐ No milk used
cereal:	☐ A lot	☐ Average	☐ Hardly any	☐ No milk used

3. *Did you drink decaffeinated tea or coffee?*

Tea:	☐ Always	☐ Sometimes	☐ Never
Coffee:	☐ Always	☐ Sometimes	☐ Never

4. *Which types of fat did you use last week for baking, frying, spreading and on salads?* **If you are not sure which category to indicate, check packaging for the exact name, fat content and brand and fill in this information**

Type of fat, spread or margarine	Brand and name or product	Spreading	Frying	Baking	Salads
Butter					
Spreadable butter					
Diary spread (e.g. I can't believe it's not butter)					
Polyunsaturated spread (sunflower, soya or vegan)					
Low fat spread (less than 60% fat)					

Type of fat, spread or margarine	Brand and name or product	Spreading	Frying	Baking	Salads
Olive oil based spread					
Other soft margarine or spread - 1					
Other soft margarine or spread - 2					
Hard margarine					
Vegetable oil - 1					
Vegetable oil - 2					
Lard					
White vegetable fat					
Dripping or animal fat					
Other					

5. *Which type of bread did you eat most often last week?*
Select one only.

☐ White ☐ Soft grain
☐ Granary ☐ Brown
☐ Wholemeal ☐ wheatgerm

Other:

6. *Did you eat butter, margarine or spread last week?*
Please tick boxes below to show whether you ate it on toast, bread, sandwiches, in rolls or on crackers:

	Toast	Bread	Sandwiches	Rolls	Crackers
Always					
Sometimes					
Never					
Don't know					

7. *How thickly did you spread butter, margarine etc. on bread or crackers?*

☐ Thick ☐ Medium
☐ Thin ☐ None

8. *If you ate grilled, fried, barbecued or roast meat last week, how well cooked was it?*
Please tick the boxes.

	Red meat: e.g. Beef, lamb	Poultry
Well done or dark brown		
Medium		
Lightly cooked or rare		
Did not eat meats cooked by these methods		
Did not eat these meats		

9. *If you ate red meat last week, what did you do with the visible fat?*
Please note that red meat includes beef, lamb, etc.

☐ Ate all of the fat ☐ Ate most of the fat
☐ Ate some of the fat ☐ Ate as little as possible
☐ Did not eat meat ☐ No fat eaten

10. *If you ate poultry last week, did you eat the skin?* ***Please note that poultry includes chicken, duck, goose and game birds.***

☐ Yes ☐ No
☐ Sometimes ☐ Did not eat poultry

11. *If you had gravy last week, were the meat juices, pan residues or dripping put into the gravy?*

☐ Yes ☐ No ☐ Sometimes
☐ Don't know ☐ Did not eat gravy

12. *Was salt usually added to your food during cooking last week?*

☐ Yes ☐ No ☐ Don't know

Did you usually add salt to your food at the table last week?

☐ Yes ☐ No ☐ Don't know

Did you regularly use a salt substitute (e.g. LoSalt) last week?

☐ Yes ☐ No ☐ Don't know

13. *Did you eat the skin on fruit?* ***Please tick boxes.***

	Apple	Pear
Skin eaten		
Skin not eaten		
Fruit not eaten		

14. *Please name any vitamins, minerals or other food supplements taken on each day of last week.* ***Please write down all the details from each packet/container or enclose label(s). Give the number of tablets taken on each day.***

Example

Name	Strength	capsule	Tablet tsp.	Day 1	Day 2	Day 3
Boots	High strength vitamin C	1000 mg	Tablet	1	1	2

15. *Which types of water did you consume last week?*
Please give information for both HOT and COLD drinks.

Water type	Hot drink	Cold drink
Tap water (unfiltered)		
Filtered water – hard water filter		
Filtered water – other		
Bottled water – *brand:*		
Other water – *brand:*		

16. *Were any of the following foods which you ate last week produced* ***organically*** *(without pesticides)?* ***Please tick the necessary box(es).***

- ☐ Vegetables, homegrown
- ☐ vegetables, purchased
- ☐ Fruits, homegrown
- ☐ Fruit, purchased
- ☐ Milk and diary products
- ☐ cereal or cereal products, bread
- ☐ Meat
- ☐ No organic foods eaten

**This space has been left for you to tell us about anything else which you feel is important about your food/drink intake in last week.**

Please bring the diary back with you to the clinic for your health screening appointment.

Thank you very much for your help

in completing such a detailed record
