# Supplementary material for: The dietary changes during Ramadan and their impact on anthropometry, blood pressure, and metabolic profile
Source: Front Nutr. 2024 Jun 10;11:1394673. doi: 10.3389/fnut.2024.1394673 (PMC11194389; doi:10.3389/fnut.2024.1394673)
Supplement: Supplementary file 1 [file Data_Sheet_1.zip › Supplementary Material 2.DOCX]

Supplementary Material 2: Changes in metabolites after RIF in males (n=20).

| **metabolite** | **Mean difference (95% CI)** | **FDR adjusted p-value** |
| --- | --- | --- |
| Lactate | -0.237 (-0.07 to 0.05) | 0.005 |
| Acetate | -0.163 (-0.08 to 0.04) | 0.629 |
| Total_C | 0.031 (-0.04 to 0.08) | 0.944 |
| non_HDL_C | 0.027 (-0.04 to 0.09) | 0.944 |
| Remnant_C | 0.009 (-0.09 to 0.06) | 0.944 |
| VLDL_C | -0.011 (-0.02 to 0.13) | 0.944 |
| Clinical_LDL_C | 0.042 (-0.04 to 0.07) | 0.944 |
| LDL_C | 0.044 (-0.05 to 0.08) | 0.944 |
| HDL_C | 0.017 (-0.08 to 0.06) | 0.944 |
| Total_TG | -0.02 (-0.03 to 0.1) | 0.944 |
| VLDL_TG | -0.007 (-0.06 to 0.06) | 0.944 |
| LDL_TG | -0.06 (-0.05 to 0.06) | 0.944 |
| HDL_TG | -0.072 (-0.09 to 0.07) | 0.944 |
| Total_PL | 0.006 (-0.04 to 0.1) | 0.944 |
| VLDL_PL | -0.017 (-0.06 to 0.06) | 0.944 |
| LDL_PL | 0.035 (-0.07 to 0.05) | 0.944 |
| Total_CE | 0.033 (-0.05 to 0.07) | 0.944 |
| VLDL_CE | -0.009 (-0.08 to 0.05) | 0.944 |
| LDL_CE | 0.04 (-0.09 to 0.05) | 0.944 |
| HDL_CE | 0.018 (-0.07 to 0.05) | 0.944 |
| Total_FC | 0.023 (-0.06 to 0.07) | 0.944 |
| VLDL_FC | -0.014 (-0.04 to 0.07) | 0.944 |
| LDL_FC | 0.055 (-0.05 to 0.09) | 0.944 |
| HDL_FC | 0.013 (-0.06 to 0.05) | 0.944 |
| Total_L | 0.011 (-0.06 to 0.1) | 0.944 |
| VLDL_L | -0.01 (-0.09 to 0.04) | 0.944 |
| LDL_L | 0.034 (-0.09 to 0.07) | 0.944 |
| Total_P | 0.005 (-0.1 to 0.04) | 0.944 |
| VLDL_P | -0.013 (-0.1 to 0.05) | 0.944 |
| LDL_P | 0.027 (-0.11 to 0.04) | 0.944 |
| VLDL_size | -0.011 (-0.1 to 0.04) | 0.944 |
| LDL_size | 0.033 (-0.04 to 0.13) | 0.944 |
| HDL_size | 0.009 (-0.19 to 0.04) | 0.944 |
| Phosphoglyc | -0.012 (-0.06 to 0.14) | 0.944 |
| TG_by_PG | -0.023 (-0.09 to 0.06) | 0.944 |
| Cholines | -0.007 (-0.16 to 0.03) | 0.944 |
| Phosphatidylc | 0.006 (-0.19 to 0.01) | 0.944 |
| Sphingomyelins | 0.012 (-0.16 to 0.04) | 0.944 |
| ApoB | 0.018 (-0.22 to 0) | 0.944 |
| ApoA1 | -0.006 (-0.18 to 0.01) | 0.944 |
| ApoB_by_ApoA1 | 0.02 (-0.26 to 0.01) | 0.944 |
| Total_FA | -0.027 (-0.17 to 0.02) | 0.944 |
| Unsaturation | -0.013 (-0.09 to 0.11) | 0.944 |
| Omega_3 | 0.016 (-0.33 to -0.14) | 0.944 |
| Omega_6 | -0.03 (-0.15 to 0) | 0.944 |
| PUFA | -0.026 (-0.13 to 0.08) | 0.944 |
| MUFA | -0.038 (-0.14 to 0.11) | 0.944 |
| SFA | -0.016 (-0.27 to -0.06) | 0.944 |
| LA | -0.031 (-0.13 to 0.05) | 0.944 |
| DHA | 0.047 (-0.03 to 0.23) | 0.944 |
| Ala | -0.073 (-0.04 to 0.04) | 0.944 |
| Gln | 0.043 (-0.13 to 0.03) | 0.944 |
| Gly | -0.016 (-0.18 to -0.02) | 0.944 |
| His | -0.063 (-0.1 to 0.03) | 0.944 |
| Total_BCAA | -0.088 (-0.09 to 0.03) | 0.944 |
| Ile | -0.058 (-0.11 to 0.01) | 0.944 |
| Leu | -0.11 (-0.11 to 0.01) | 0.944 |
| Val | -0.088 (-0.11 to 0.01) | 0.944 |
| Phe | -0.126 (-0.11 to 0.01) | 0.944 |
| Tyr | -0.072 (-0.08 to 0.04) | 0.944 |
| Glucose | 0.008 (-0.08 to 0.04) | 0.944 |
| Pyruvate | -0.075 (-0.08 to 0.04) | 0.944 |
| Citrate | -0.024 (-0.08 to 0.05) | 0.944 |
| bOHbutyrate | -0.017 (-0.08 to 0.06) | 0.944 |
| Acetoacetate | -0.042 (-0.07 to 0.06) | 0.944 |
| Acetone | 0.1 (-0.08 to 0.06) | 0.944 |
| Creatinine | 0.004 (-0.07 to 0.06) | 0.944 |
| Albumin | -0.052 (-0.09 to 0.05) | 0.944 |
| GlycA | -0.1 (-0.09 to 0.06) | 0.944 |
| XXL_VLDL_P | -0.036 (-0.1 to 0.07) | 0.944 |
| XXL_VLDL_L | -0.03 (-0.08 to 0.05) | 0.944 |
| XXL_VLDL_PL | -0.049 (-0.06 to 0.08) | 0.944 |
| XXL_VLDL_C | -0.049 (-0.06 to 0.09) | 0.944 |
| XXL_VLDL_CE | -0.049 (-0.07 to 0.09) | 0.944 |
| XXL_VLDL_FC | -0.049 (-0.07 to 0.09) | 0.944 |
| XXL_VLDL_TG | -0.017 (-0.05 to 0.1) | 0.944 |
| XL_VLDL_P | -0.018 (-0.05 to 0.11) | 0.944 |
| XL_VLDL_L | -0.012 (-0.06 to 0.09) | 0.944 |
| XL_VLDL_PL | -0.018 (-0.07 to 0.08) | 0.944 |
| XL_VLDL_C | -0.017 (-0.11 to 0.08) | 0.944 |
| XL_VLDL_CE | -0.012 (-0.1 to 0.07) | 0.944 |
| XL_VLDL_FC | -0.021 (-0.08 to 0.07) | 0.944 |
| XL_VLDL_TG | -0.008 (-0.09 to 0.06) | 0.944 |
| L_VLDL_P | -0.009 (-0.1 to 0.05) | 0.944 |
| L_VLDL_PL | -0.016 (-0.12 to 0.07) | 0.944 |
| L_VLDL_C | -0.015 (-0.09 to 0.04) | 0.944 |
| L_VLDL_CE | -0.014 (-0.09 to 0.04) | 0.944 |
| L_VLDL_FC | -0.015 (-0.1 to 0.04) | 0.944 |
| L_VLDL_TG | 0.013 (-0.07 to 0.07) | 0.944 |
| M_VLDL_P | 0.014 (-0.07 to 0.07) | 0.944 |
| M_VLDL_L | 0.014 (-0.09 to 0.05) | 0.944 |
| M_VLDL_PL | 0.011 (-0.14 to 0.02) | 0.944 |
| M_VLDL_C | 0.024 (-0.06 to 0.07) | 0.944 |
| M_VLDL_CE | 0.031 (-0.04 to 0.08) | 0.944 |
| M_VLDL_FC | 0.016 (-0.04 to 0.09) | 0.944 |
| M_VLDL_TG | 0.005 (-0.03 to 0.1) | 0.944 |
| S_VLDL_P | -0.015 (-0.03 to 0.1) | 0.944 |
| S_VLDL_L | -0.017 (-0.04 to 0.09) | 0.944 |
| S_VLDL_PL | -0.005 (-0.13 to 0.01) | 0.944 |
| S_VLDL_C | -0.016 (-0.05 to 0.11) | 0.944 |
| S_VLDL_CE | -0.027 (-0.03 to 0.11) | 0.944 |
| S_VLDL_FC | 0.006 (-0.04 to 0.1) | 0.944 |
| S_VLDL_TG | -0.021 (-0.02 to 0.13) | 0.944 |
| XS_VLDL_P | -0.026 (-0.02 to 0.12) | 0.944 |
| XS_VLDL_L | -0.023 (-0.02 to 0.12) | 0.944 |
| XS_VLDL_PL | -0.027 (-0.12 to 0.01) | 0.944 |
| XS_VLDL_CE | 0.005 (-0.04 to 0.09) | 0.944 |
| XS_VLDL_FC | -0.02 (-0.04 to 0.09) | 0.944 |
| XS_VLDL_TG | -0.062 (-0.04 to 0.09) | 0.944 |
| IDL_L | 0.021 (-0.02 to 0.13) | 0.944 |
| IDL_PL | 0.022 (-0.13 to 0.01) | 0.944 |
| IDL_C | 0.032 (-0.04 to 0.09) | 0.944 |
| IDL_CE | 0.032 (-0.03 to 0.09) | 0.944 |
| IDL_FC | 0.028 (-0.01 to 0.13) | 0.944 |
| IDL_TG | -0.058 (-0.04 to 0.09) | 0.944 |
| L_LDL_P | 0.031 (-0.05 to 0.07) | 0.944 |
| L_LDL_L | 0.041 (0.01 to 0.16) | 0.944 |
| L_LDL_PL | 0.032 (-0.11 to 0.02) | 0.944 |
| L_LDL_C | 0.054 (-0.03 to 0.06) | 0.944 |
| L_LDL_CE | 0.055 (-0.04 to 0.07) | 0.944 |
| L_LDL_FC | 0.049 (-0.04 to 0.07) | 0.944 |
| L_LDL_TG | -0.059 (-0.03 to 0.08) | 0.944 |
| M_LDL_P | 0.018 (-0.03 to 0.08) | 0.944 |
| M_LDL_L | 0.022 (-0.04 to 0.08) | 0.944 |
| M_LDL_PL | 0.029 (-0.1 to 0.03) | 0.944 |
| M_LDL_C | 0.027 (-0.04 to 0.07) | 0.944 |
| M_LDL_CE | 0.017 (-0.04 to 0.08) | 0.944 |
| M_LDL_FC | 0.054 (-0.05 to 0.08) | 0.944 |
| M_LDL_TG | -0.06 (-0.04 to 0.08) | 0.944 |
| S_LDL_P | 0.022 (-0.04 to 0.09) | 0.944 |
| S_LDL_L | 0.031 (-0.04 to 0.08) | 0.944 |
| S_LDL_PL | 0.059 (-0.11 to 0.02) | 0.944 |
| S_LDL_C | 0.029 (-0.07 to 0.06) | 0.944 |
| S_LDL_CE | 0.011 (-0.08 to 0.06) | 0.944 |
| S_LDL_FC | 0.082 (-0.09 to 0.06) | 0.944 |
| S_LDL_TG | -0.043 (-0.06 to 0.07) | 0.944 |
| XL_HDL_P | 0.015 (-0.06 to 0.07) | 0.944 |
| XL_HDL_L | 0.017 (-0.05 to 0.07) | 0.944 |
| XL_HDL_PL | 0.013 (-0.17 to 0.02) | 0.944 |
| XL_HDL_C | 0.023 (-0.08 to 0.07) | 0.944 |
| XL_HDL_CE | 0.024 (-0.1 to 0.06) | 0.944 |
| XL_HDL_FC | 0.02 (-0.1 to 0.05) | 0.944 |
| XL_HDL_TG | -0.034 (-0.07 to 0.08) | 0.944 |
| L_HDL_P | 0.016 (-0.06 to 0.07) | 0.944 |
| L_HDL_L | 0.017 (-0.08 to 0.06) | 0.944 |
| L_HDL_PL | 0.018 (-0.16 to 0.02) | 0.944 |
| L_HDL_C | 0.022 (-0.08 to 0.06) | 0.944 |
| L_HDL_CE | 0.023 (-0.08 to 0.06) | 0.944 |
| L_HDL_FC | 0.02 (-0.08 to 0.06) | 0.944 |
| L_HDL_TG | -0.047 (-0.08 to 0.06) | 0.944 |
| M_HDL_P | -0.006 (-0.08 to 0.06) | 0.944 |
| M_HDL_L | -0.01 (-0.08 to 0.06) | 0.944 |
| M_HDL_PL | -0.014 (-0.08 to 0.06) | 0.944 |
| M_HDL_C | 0.005 (-0.08 to 0.06) | 0.944 |
| M_HDL_CE | 0.005 (-0.08 to 0.06) | 0.944 |
| M_HDL_FC | 0.007 (-0.08 to 0.06) | 0.944 |
| M_HDL_TG | -0.077 (-0.08 to 0.06) | 0.944 |
| S_HDL_L | -0.022 (-0.08 to 0.06) | 0.944 |
| S_HDL_PL | -0.026 (-0.08 to 0.06) | 0.944 |
| S_HDL_CE | 0.007 (-0.08 to 0.06) | 0.944 |
| S_HDL_FC | -0.011 (-0.08 to 0.06) | 0.944 |
| S_HDL_TG | -0.069 (-0.08 to 0.06) | 0.944 |
| HDL_L | 0.002 (-0.09 to 0.13) | 0.957 |
| XS_VLDL_C | -0.003 (-0.05 to 0.09) | 0.957 |
| IDL_P | 0.003 (-0.05 to 0.08) | 0.957 |
| S_HDL_P | -0.003 (-0.08 to 0.06) | 0.957 |
| S_HDL_C | 0.004 (-0.08 to 0.06) | 0.957 |
| HDL_P | 0.002 (-0.07 to 0.04) | 0.961 |
| HDL_PL | -0.002 (-0.1 to 0.17) | 0.966 |
| L_VLDL_L | -0.001 (-0.07 to 0.08) | 0.97 |
