# Supplementary material for: The dietary changes during Ramadan and their impact on anthropometry, blood pressure, and metabolic profile
Source: Front Nutr. 2024 Jun 10;11:1394673. doi: 10.3389/fnut.2024.1394673 (PMC11194389; doi:10.3389/fnut.2024.1394673)
Supplement: Supplementary file 1 [file Data_Sheet_1.zip › Supplementary Material 3.DOCX]

Supplementary Material 3: Changes in metabolites after RIF in females (n=25).

| **metabolite** | **Mean difference (95% CI)** | **FDR adjusted p-value** |
| --- | --- | --- |
| Lactate | -0.34 (-0.04 to 0.05) | <0.001 |
| Acetate | -0.301 (-0.05 to 0.04) | <0.001 |
| HDL_PL | -0.12 (-0.11 to 0.03) | 0.006 |
| L_HDL_P | -0.061 (-0.16 to 0) | 0.006 |
| L_HDL_PL | -0.073 (-0.11 to -0.01) | 0.006 |
| HDL_L | -0.111 (-0.16 to -0.02) | 0.006 |
| L_HDL_L | -0.065 (-0.21 to -0.05) | 0.006 |
| M_HDL_PL | -0.137 (-0.11 to -0.01) | 0.006 |
| Phosphatidylc | -0.109 (-0.22 to -0.04) | 0.007 |
| Phosphoglyc | -0.122 (-0.01 to 0.12) | 0.007 |
| M_HDL_L | -0.131 (-0.11 to -0.01) | 0.007 |
| HDL_FC | -0.093 (-0.18 to -0.06) | 0.007 |
| ApoA1 | -0.122 (-0.2 to -0.03) | 0.007 |
| Cholines | -0.119 (-0.13 to 0.01) | 0.007 |
| M_HDL_P | -0.124 (-0.11 to -0.01) | 0.007 |
| L_HDL_FC | -0.053 (-0.11 to -0.01) | 0.007 |
| M_HDL_FC | -0.113 (-0.11 to -0.01) | 0.009 |
| XS_VLDL_CE | -0.08 (-0.11 to 0.01) | 0.009 |
| Total_PL | -0.115 (-0.09 to 0.01) | 0.009 |
| L_LDL_TG | -0.071 (-0.04 to 0) | 0.009 |
| XS_VLDL_C | -0.075 (-0.09 to 0) | 0.01 |
| IDL_TG | -0.071 (-0.1 to 0.02) | 0.01 |
| Tyr | -0.195 (-0.03 to 0.07) | 0.01 |
| L_HDL_C | -0.054 (-0.11 to -0.01) | 0.01 |
| XS_VLDL_P | -0.076 (-0.13 to 0.01) | 0.01 |
| L_HDL_TG | -0.081 (-0.11 to -0.01) | 0.01 |
| S_HDL_PL | -0.129 (-0.11 to -0.01) | 0.011 |
| XS_VLDL_L | -0.072 (-0.14 to 0.01) | 0.011 |
| L_HDL_CE | -0.054 (-0.11 to -0.01) | 0.011 |
| M_HDL_TG | -0.104 (-0.11 to -0.01) | 0.011 |
| M_HDL_C | -0.116 (-0.11 to -0.01) | 0.013 |
| HDL_C | -0.092 (-0.06 to 0.03) | 0.013 |
| LDL_TG | -0.069 (-0.19 to -0.05) | 0.014 |
| XS_VLDL_FC | -0.066 (-0.12 to 0.01) | 0.014 |
| IDL_PL | -0.081 (-0.11 to -0.02) | 0.014 |
| HDL_size | -0.04 (-0.2 to -0.05) | 0.014 |
| M_HDL_CE | -0.115 (-0.11 to -0.01) | 0.014 |
| Ala | -0.122 (-0.08 to 0) | 0.014 |
| XS_VLDL_PL | -0.065 (-0.11 to -0.03) | 0.015 |
| IDL_L | -0.091 (-0.13 to 0.03) | 0.016 |
| IDL_P | -0.092 (-0.11 to 0) | 0.016 |
| HDL_CE | -0.091 (-0.18 to -0.06) | 0.016 |
| HDL_TG | -0.092 (-0.1 to 0) | 0.016 |
| Total_P | -0.12 (-0.15 to -0.03) | 0.016 |
| HDL_P | -0.122 (-0.1 to -0.01) | 0.016 |
| PUFA | -0.101 (-0.09 to 0.08) | 0.016 |
| S_HDL_FC | -0.129 (-0.11 to -0.01) | 0.016 |
| Unsaturation | -0.116 (-0.02 to 0.1) | 0.017 |
| Leu | -0.155 (-0.06 to 0.04) | 0.017 |
| S_HDL_L | -0.125 (-0.11 to -0.01) | 0.018 |
| Omega_6 | -0.093 (-0.17 to -0.02) | 0.018 |
| Acetone | 0.162 (-0.04 to 0.05) | 0.019 |
| LA | -0.083 (-0.28 to -0.03) | 0.02 |
| GlycA | -0.09 (-0.06 to 0.04) | 0.02 |
| Total_BCAA | -0.127 (-0.04 to 0.06) | 0.021 |
| IDL_C | -0.09 (-0.07 to 0.03) | 0.022 |
| IDL_CE | -0.088 (-0.09 to 0.02) | 0.022 |
| Total_L | -0.086 (0 to 0.06) | 0.023 |
| Total_FA | -0.073 (-0.3 to -0.09) | 0.024 |
| XL_HDL_P | -0.03 (-0.18 to -0.05) | 0.024 |
| IDL_FC | -0.087 (-0.07 to 0.04) | 0.026 |
| M_LDL_TG | -0.064 (-0.08 to -0.02) | 0.026 |
| Remnant_C | -0.074 (-0.09 to 0.02) | 0.027 |
| Sphingomyelins | -0.103 (-0.21 to -0.03) | 0.027 |
| Total_FC | -0.084 (-0.16 to -0.06) | 0.029 |
| Total_C | -0.094 (-0.15 to -0.04) | 0.03 |
| Total_CE | -0.094 (-0.06 to -0.02) | 0.03 |
| Ile | -0.118 (-0.05 to 0.04) | 0.03 |
| S_VLDL_CE | -0.061 (-0.13 to 0) | 0.03 |
| Val | -0.113 (-0.06 to 0.04) | 0.034 |
| SFA | -0.056 (-0.39 to -0.21) | 0.035 |
| XS_VLDL_TG | -0.066 (-0.11 to 0.01) | 0.035 |
| Pyruvate | -0.099 (-0.04 to 0.05) | 0.04 |
| Phe | -0.115 (-0.06 to 0.03) | 0.041 |
| Omega_3 | -0.09 (-0.43 to -0.25) | 0.043 |
| S_VLDL_C | -0.059 (-0.09 to 0.01) | 0.043 |
| Albumin | -0.115 (-0.05 to 0.05) | 0.044 |
| XL_HDL_CE | -0.026 (-0.21 to -0.05) | 0.046 |
| Acetoacetate | -0.154 (-0.03 to 0.06) | 0.052 |
| S_HDL_C | -0.112 (-0.11 to -0.01) | 0.055 |
| M_VLDL_CE | -0.069 (-0.15 to -0.04) | 0.055 |
| S_HDL_P | -0.106 (-0.11 to -0.01) | 0.055 |
| S_HDL_TG | -0.059 (-0.11 to -0.01) | 0.055 |
| XL_HDL_TG | -0.055 (-0.21 to -0.02) | 0.057 |
| VLDL_CE | -0.058 (-0.18 to -0.06) | 0.058 |
| MUFA | -0.057 (-0.05 to 0.16) | 0.059 |
| DHA | -0.075 (0.05 to 0.27) | 0.064 |
| non_HDL_C | -0.065 (-0.14 to -0.02) | 0.066 |
| XL_HDL_L | -0.025 (-0.17 to -0.05) | 0.067 |
| L_LDL_PL | -0.076 (-0.09 to 0) | 0.079 |
| S_VLDL_FC | -0.055 (-0.15 to -0.01) | 0.082 |
| S_HDL_CE | -0.082 (-0.11 to -0.01) | 0.085 |
| M_VLDL_C | -0.058 (-0.15 to -0.04) | 0.093 |
| S_VLDL_PL | -0.053 (-0.11 to -0.03) | 0.093 |
| XL_HDL_PL | -0.025 (-0.16 to -0.04) | 0.093 |
| XL_HDL_C | -0.021 (-0.2 to -0.02) | 0.096 |
| Creatinine | -0.043 (-0.04 to 0.05) | 0.098 |
| ApoB | -0.049 (-0.26 to -0.05) | 0.106 |
| VLDL_C | -0.049 (-0.14 to 0.02) | 0.112 |
| L_LDL_L | -0.066 (-0.09 to 0.05) | 0.116 |
| M_LDL_P | -0.045 (-0.05 to -0.01) | 0.125 |
| LDL_L | -0.059 (-0.19 to -0.04) | 0.133 |
| ApoB_by_ApoA1 | 0.03 (-0.2 to -0.03) | 0.133 |
| L_LDL_FC | -0.067 (-0.05 to 0) | 0.133 |
| S_LDL_TG | -0.041 (-0.18 to -0.05) | 0.133 |
| LDL_PL | -0.061 (-0.02 to 0.06) | 0.133 |
| M_LDL_CE | -0.052 (-0.1 to -0.03) | 0.133 |
| VLDL_P | -0.048 (-0.16 to -0.04) | 0.135 |
| Gln | 0.059 (-0.2 to -0.02) | 0.14 |
| Clinical_LDL_C | -0.061 (-0.14 to -0.05) | 0.142 |
| S_LDL_CE | -0.047 (-0.19 to -0.07) | 0.147 |
| LDL_CE | -0.055 (-0.02 to 0.08) | 0.149 |
| S_VLDL_L | -0.047 (-0.15 to -0.03) | 0.149 |
| M_LDL_L | -0.052 (-0.03 to 0.02) | 0.149 |
| LDL_C | -0.057 (-0.14 to -0.03) | 0.158 |
| His | -0.057 (-0.04 to 0.05) | 0.158 |
| L_LDL_C | -0.061 (-0.05 to -0.01) | 0.158 |
| M_LDL_C | -0.051 (-0.09 to -0.03) | 0.158 |
| L_LDL_CE | -0.057 (-0.05 to 0) | 0.171 |
| LDL_FC | -0.06 (-0.1 to 0) | 0.195 |
| M_VLDL_FC | -0.041 (-0.13 to -0.03) | 0.195 |
| M_LDL_PL | -0.051 (-0.1 to -0.01) | 0.195 |
| LDL_P | -0.038 (-0.1 to -0.01) | 0.207 |
| M_VLDL_PL | -0.039 (-0.11 to -0.02) | 0.218 |
| S_LDL_C | -0.042 (-0.19 to -0.06) | 0.218 |
| L_LDL_P | -0.038 (-0.1 to 0.01) | 0.229 |
| VLDL_FC | -0.035 (-0.18 to -0.03) | 0.241 |
| S_VLDL_P | -0.038 (-0.15 to -0.03) | 0.264 |
| S_LDL_L | -0.036 (-0.08 to -0.03) | 0.264 |
| M_VLDL_P | -0.035 (-0.12 to -0.04) | 0.288 |
| VLDL_PL | -0.031 (-0.2 to -0.05) | 0.296 |
| LDL_size | -0.042 (-0.14 to -0.01) | 0.296 |
| M_LDL_FC | -0.048 (-0.11 to -0.04) | 0.307 |
| Glucose | 0.036 (-0.04 to 0.05) | 0.317 |
| Gly | -0.04 (-0.15 to -0.03) | 0.327 |
| TG_by_PG | 0.029 (-0.11 to 0.03) | 0.35 |
| bOHbutyrate | 0.054 (-0.07 to 0.03) | 0.397 |
| L_VLDL_TG | 0.024 (-0.12 to -0.04) | 0.407 |
| M_VLDL_L | -0.027 (-0.11 to -0.03) | 0.416 |
| XXL_VLDL_TG | 0.022 (-0.11 to 0) | 0.449 |
| VLDL_size | 0.02 (-0.14 to -0.03) | 0.47 |
| XL_VLDL_TG | 0.018 (-0.11 to -0.01) | 0.523 |
| S_LDL_P | -0.02 (-0.08 to -0.02) | 0.523 |
| L_VLDL_CE | -0.021 (-0.11 to -0.03) | 0.543 |
| VLDL_L | -0.017 (-0.12 to -0.02) | 0.571 |
| XL_VLDL_CE | -0.018 (-0.1 to 0.01) | 0.571 |
| S_VLDL_TG | -0.02 (-0.13 to 0.01) | 0.601 |
| S_LDL_FC | -0.022 (-0.2 to -0.07) | 0.632 |
| XXL_VLDL_FC | -0.014 (-0.09 to 0.01) | 0.646 |
| S_LDL_PL | -0.015 (-0.13 to -0.04) | 0.646 |
| Total_TG | -0.011 (-0.12 to 0) | 0.705 |
| XXL_VLDL_C | -0.012 (-0.09 to 0.02) | 0.705 |
| L_VLDL_C | -0.012 (-0.12 to -0.03) | 0.722 |
| XXL_VLDL_L | 0.01 (-0.05 to 0.04) | 0.757 |
| XL_VLDL_C | -0.009 (-0.09 to 0.02) | 0.757 |
| XXL_VLDL_CE | -0.01 (-0.08 to 0.03) | 0.763 |
| XL_VLDL_L | 0.009 (-0.09 to 0.01) | 0.769 |
| L_VLDL_L | 0.008 (-0.1 to 0) | 0.77 |
| XL_HDL_FC | -0.005 (-0.2 to -0.06) | 0.77 |
| VLDL_TG | 0.007 (-0.16 to -0.06) | 0.829 |
| XXL_VLDL_PL | -0.005 (-0.02 to 0.07) | 0.876 |
| XL_VLDL_PL | 0.003 (-0.05 to 0.05) | 0.918 |
| L_VLDL_FC | -0.004 (-0.1 to -0.03) | 0.918 |
| XXL_VLDL_P | 0.003 (-0.08 to 0.03) | 0.923 |
| XL_VLDL_P | 0.003 (-0.13 to -0.01) | 0.927 |
| L_VLDL_P | 0.002 (-0.11 to -0.02) | 0.942 |
| XL_VLDL_FC | -0.002 (-0.1 to 0) | 0.957 |
| Citrate | -0.002 (-0.06 to 0.04) | 0.971 |
| M_VLDL_TG | 0.001 (-0.15 to -0.03) | 0.976 |
| L_VLDL_PL | 0 (-0.08 to 0.04) | 1 |
