# Supplementary material for: The dietary changes during Ramadan and their impact on anthropometry, blood pressure, and metabolic profile
Source: Front Nutr. 2024 Jun 10;11:1394673. doi: 10.3389/fnut.2024.1394673 (PMC11194389; doi:10.3389/fnut.2024.1394673)
Supplement: Supplementary file 1 [file Data_Sheet_1.zip › Supplementary Material 4.DOCX]

Supplementary Material 4: Changes in metabolites after RIF in healthy individuals (n=36).

| **metabolite** | **Mean difference (95% CI)** | **FDR adjusted p-value** |
| --- | --- | --- |
| Total_C | -0.043 (-0.09 to 0) | 0.323 |
| non_HDL_C | -0.028 (-0.09 to 0.01) | 0.486 |
| Remnant_C | -0.042 (-0.07 to 0.02) | 0.239 |
| VLDL_C | -0.035 (-0.08 to 0.05) | 0.309 |
| Clinical_LDL_C | -0.023 (-0.09 to 0) | 0.663 |
| LDL_C | -0.015 (-0.09 to 0.01) | 0.751 |
| HDL_C | -0.046 (-0.05 to 0.03) | 0.17 |
| Total_TG | -0.007 (-0.08 to 0.03) | 0.839 |
| VLDL_TG | 0.009 (-0.11 to -0.02) | 0.815 |
| LDL_TG | -0.06 (-0.13 to -0.01) | 0.062 |
| HDL_TG | -0.072 (-0.08 to 0.02) | 0.078 |
| Total_PL | -0.064 (-0.06 to 0.04) | 0.078 |
| VLDL_PL | -0.023 (-0.13 to -0.01) | 0.533 |
| LDL_PL | -0.024 (-0.02 to 0.06) | 0.607 |
| HDL_PL | -0.069 (-0.09 to 0.07) | 0.077 |
| Total_CE | -0.042 (-0.04 to 0.03) | 0.336 |
| VLDL_CE | -0.04 (-0.12 to -0.02) | 0.233 |
| LDL_CE | -0.015 (-0.03 to 0.06) | 0.751 |
| HDL_CE | -0.046 (-0.12 to -0.02) | 0.186 |
| Total_FC | -0.041 (-0.1 to 0) | 0.309 |
| VLDL_FC | -0.026 (-0.12 to 0) | 0.46 |
| LDL_FC | -0.015 (-0.07 to 0.02) | 0.783 |
| HDL_FC | -0.044 (-0.12 to -0.02) | 0.145 |
| Total_L | -0.043 (-0.03 to 0.07) | 0.26 |
| VLDL_L | -0.01 (-0.1 to 0) | 0.789 |
| LDL_L | -0.021 (-0.15 to -0.04) | 0.663 |
| HDL_L | -0.062 (-0.1 to 0.05) | 0.078 |
| Total_P | -0.071 (-0.12 to -0.02) | 0.078 |
| VLDL_P | -0.032 (-0.13 to -0.01) | 0.401 |
| LDL_P | -0.011 (-0.09 to 0) | 0.797 |
| HDL_P | -0.074 (-0.07 to 0) | 0.078 |
| VLDL_size | 0.018 (-0.11 to -0.01) | 0.607 |
| LDL_size | -0.009 (-0.07 to 0.05) | 0.876 |
| HDL_size | -0.008 (-0.18 to -0.02) | 0.796 |
| Phosphoglyc | -0.072 (0.01 to 0.12) | 0.078 |
| TG_by_PG | 0.014 (-0.08 to 0.04) | 0.738 |
| Cholines | -0.069 (-0.09 to 0.02) | 0.078 |
| Phosphatidylc | -0.052 (-0.2 to -0.05) | 0.15 |
| Sphingomyelins | -0.06 (-0.19 to -0.04) | 0.146 |
| ApoB | -0.022 (-0.23 to -0.06) | 0.578 |
| ApoA1 | -0.073 (-0.18 to -0.04) | 0.072 |
| ApoB_by_ApoA1 | 0.02 (-0.21 to -0.04) | 0.607 |
| Total_FA | -0.051 (-0.26 to -0.08) | 0.123 |
| Unsaturation | -0.095 (-0.04 to 0.09) | 0.062 |
| Omega_3 | -0.027 (-0.38 to -0.24) | 0.666 |
| Omega_6 | -0.07 (-0.17 to -0.04) | 0.078 |
| PUFA | -0.069 (-0.13 to 0.03) | 0.078 |
| MUFA | -0.044 (-0.08 to 0.11) | 0.196 |
| SFA | -0.035 (-0.26 to -0.12) | 0.186 |
| LA | -0.059 (-0.21 to -0.02) | 0.078 |
| DHA | -0.009 (0.02 to 0.23) | 0.839 |
| Ala | -0.1 (-0.07 to 0) | 0.078 |
| Gln | 0.068 (-0.16 to -0.02) | 0.078 |
| Gly | -0.017 (-0.16 to -0.06) | 0.751 |
| His | -0.034 (-0.05 to 0.03) | 0.447 |
| Total_BCAA | -0.122 (-0.05 to 0.04) | 0.058 |
| Ile | -0.112 (-0.07 to 0.02) | 0.062 |
| Leu | -0.148 (-0.07 to 0.01) | 0.044 |
| Val | -0.109 (-0.07 to 0.01) | 0.062 |
| Phe | -0.127 (-0.07 to 0.01) | 0.062 |
| Tyr | -0.167 (-0.03 to 0.05) | 0.031 |
| Glucose | 0.028 (-0.04 to 0.04) | 0.607 |
| Lactate | -0.308 (-0.03 to 0.05) | 0 |
| Pyruvate | -0.103 (-0.04 to 0.04) | 0.062 |
| Citrate | -0.05 (-0.05 to 0.03) | 0.401 |
| bOHbutyrate | 0.017 (-0.06 to 0.03) | 0.833 |
| Acetate | -0.191 (-0.05 to 0.03) | 0.001 |
| Acetoacetate | -0.117 (-0.03 to 0.06) | 0.078 |
| Acetone | 0.125 (-0.04 to 0.05) | 0.078 |
| Creatinine | -0.035 (-0.03 to 0.05) | 0.15 |
| Albumin | -0.095 (-0.04 to 0.04) | 0.078 |
| GlycA | -0.11 (-0.06 to 0.04) | 0.016 |
| XXL_VLDL_P | -0.012 (-0.07 to 0.04) | 0.751 |
| XXL_VLDL_L | -0.006 (-0.05 to 0.04) | 0.845 |
| XXL_VLDL_PL | -0.024 (-0.01 to 0.07) | 0.473 |
| XXL_VLDL_C | -0.029 (-0.07 to 0.04) | 0.369 |
| XXL_VLDL_CE | -0.028 (-0.06 to 0.05) | 0.391 |
| XXL_VLDL_FC | -0.03 (-0.07 to 0.03) | 0.336 |
| XXL_VLDL_TG | 0.008 (-0.08 to 0.02) | 0.816 |
| XL_VLDL_P | -0.001 (-0.09 to 0.02) | 0.971 |
| XL_VLDL_L | 0.006 (-0.07 to 0.03) | 0.839 |
| XL_VLDL_PL | -0.002 (-0.04 to 0.06) | 0.927 |
| XL_VLDL_C | -0.011 (-0.08 to 0.03) | 0.751 |
| XL_VLDL_CE | -0.015 (-0.08 to 0.02) | 0.715 |
| XL_VLDL_FC | -0.007 (-0.08 to 0.02) | 0.816 |
| XL_VLDL_TG | 0.016 (-0.09 to 0) | 0.663 |
| L_VLDL_P | 0.003 (-0.09 to -0.01) | 0.912 |
| L_VLDL_L | 0.012 (-0.08 to 0.02) | 0.751 |
| L_VLDL_PL | -0.001 (-0.07 to 0.05) | 0.976 |
| L_VLDL_C | -0.01 (-0.09 to -0.02) | 0.797 |
| L_VLDL_CE | -0.016 (-0.09 to -0.01) | 0.723 |
| L_VLDL_FC | -0.005 (-0.09 to -0.01) | 0.871 |
| L_VLDL_TG | 0.03 (-0.09 to 0) | 0.38 |
| M_VLDL_P | -0.015 (-0.09 to 0) | 0.751 |
| M_VLDL_L | -0.006 (-0.08 to -0.01) | 0.871 |
| M_VLDL_PL | -0.017 (-0.11 to -0.01) | 0.699 |
| M_VLDL_C | -0.029 (-0.11 to -0.01) | 0.498 |
| M_VLDL_CE | -0.036 (-0.1 to 0) | 0.411 |
| M_VLDL_FC | -0.019 (-0.09 to 0.01) | 0.663 |
| M_VLDL_TG | 0.013 (-0.09 to 0.01) | 0.751 |
| S_VLDL_P | -0.026 (-0.09 to 0.01) | 0.607 |
| S_VLDL_L | -0.032 (-0.1 to 0.01) | 0.447 |
| S_VLDL_PL | -0.033 (-0.1 to -0.02) | 0.38 |
| S_VLDL_C | -0.044 (-0.06 to 0.04) | 0.175 |
| S_VLDL_CE | -0.051 (-0.08 to 0.04) | 0.105 |
| S_VLDL_FC | -0.032 (-0.09 to 0.02) | 0.391 |
| S_VLDL_TG | -0.01 (-0.07 to 0.05) | 0.839 |
| XS_VLDL_P | -0.055 (-0.07 to 0.05) | 0.077 |
| XS_VLDL_L | -0.05 (-0.08 to 0.04) | 0.078 |
| XS_VLDL_PL | -0.047 (-0.1 to -0.03) | 0.078 |
| XS_VLDL_C | -0.045 (-0.07 to 0.03) | 0.128 |
| XS_VLDL_CE | -0.045 (-0.08 to 0.03) | 0.15 |
| XS_VLDL_FC | -0.046 (-0.07 to 0.03) | 0.08 |
| XS_VLDL_TG | -0.057 (-0.07 to 0.03) | 0.09 |
| IDL_P | -0.062 (-0.07 to 0.02) | 0.078 |
| IDL_L | -0.045 (-0.08 to 0.05) | 0.213 |
| IDL_PL | -0.039 (-0.1 to -0.01) | 0.279 |
| IDL_C | -0.041 (-0.05 to 0.04) | 0.295 |
| IDL_CE | -0.039 (-0.06 to 0.04) | 0.323 |
| IDL_FC | -0.046 (-0.03 to 0.06) | 0.239 |
| IDL_TG | -0.059 (-0.07 to 0.03) | 0.062 |
| L_LDL_P | -0.008 (-0.07 to 0.02) | 0.839 |
| L_LDL_L | -0.022 (-0.05 to 0.08) | 0.666 |
| L_LDL_PL | -0.036 (-0.08 to 0.01) | 0.439 |
| L_LDL_C | -0.012 (-0.03 to 0.02) | 0.816 |
| L_LDL_CE | -0.008 (-0.03 to 0.03) | 0.85 |
| L_LDL_FC | -0.022 (-0.04 to 0.03) | 0.697 |
| L_LDL_TG | -0.06 (-0.03 to 0.03) | 0.054 |
| M_LDL_P | -0.021 (-0.03 to 0.03) | 0.607 |
| M_LDL_L | -0.024 (-0.02 to 0.04) | 0.599 |
| M_LDL_PL | -0.02 (-0.07 to 0.01) | 0.674 |
| M_LDL_C | -0.022 (-0.06 to 0.01) | 0.626 |
| M_LDL_CE | -0.025 (-0.06 to 0.01) | 0.533 |
| M_LDL_FC | -0.01 (-0.07 to 0.01) | 0.839 |
| M_LDL_TG | -0.056 (-0.05 to 0.02) | 0.078 |
| S_LDL_P | -0.002 (-0.06 to 0.02) | 0.967 |
| S_LDL_L | -0.009 (-0.05 to 0.02) | 0.816 |
| S_LDL_PL | 0.016 (-0.1 to -0.01) | 0.707 |
| S_LDL_C | -0.015 (-0.13 to -0.02) | 0.73 |
| S_LDL_CE | -0.025 (-0.14 to -0.03) | 0.527 |
| S_LDL_FC | 0.016 (-0.14 to -0.03) | 0.751 |
| S_LDL_TG | -0.035 (-0.12 to -0.01) | 0.239 |
| XL_HDL_P | -0.005 (-0.12 to -0.01) | 0.839 |
| XL_HDL_L | -0.002 (-0.11 to -0.01) | 0.942 |
| XL_HDL_PL | -0.003 (-0.14 to -0.02) | 0.907 |
| XL_HDL_C | 0.003 (-0.14 to 0) | 0.907 |
| XL_HDL_CE | 0 (-0.16 to -0.02) | 0.99 |
| XL_HDL_FC | 0.011 (-0.15 to -0.03) | 0.699 |
| XL_HDL_TG | -0.032 (-0.15 to 0) | 0.295 |
| L_HDL_P | -0.022 (-0.12 to 0.01) | 0.425 |
| L_HDL_L | -0.025 (-0.15 to -0.01) | 0.411 |
| L_HDL_PL | -0.029 (-0.11 to 0) | 0.369 |
| L_HDL_C | -0.016 (-0.09 to 0.01) | 0.607 |
| L_HDL_CE | -0.016 (-0.09 to 0.01) | 0.626 |
| L_HDL_FC | -0.016 (-0.09 to 0.01) | 0.599 |
| L_HDL_TG | -0.054 (-0.09 to 0.01) | 0.078 |
| M_HDL_P | -0.076 (-0.09 to 0.01) | 0.072 |
| M_HDL_L | -0.081 (-0.09 to 0.01) | 0.067 |
| M_HDL_PL | -0.087 (-0.09 to 0.01) | 0.062 |
| M_HDL_C | -0.068 (-0.09 to 0.01) | 0.078 |
| M_HDL_CE | -0.069 (-0.09 to 0.01) | 0.078 |
| M_HDL_FC | -0.061 (-0.09 to 0.01) | 0.085 |
| M_HDL_TG | -0.082 (-0.09 to 0.01) | 0.078 |
| S_HDL_P | -0.073 (-0.09 to 0.01) | 0.139 |
| S_HDL_L | -0.089 (-0.09 to 0.01) | 0.078 |
| S_HDL_PL | -0.092 (-0.09 to 0.01) | 0.067 |
| S_HDL_C | -0.074 (-0.09 to 0.01) | 0.15 |
| S_HDL_CE | -0.056 (-0.09 to 0.01) | 0.198 |
| S_HDL_FC | -0.08 (-0.09 to 0.01) | 0.078 |
| S_HDL_TG | -0.057 (-0.09 to 0.01) | 0.139 |
